# Coumarin-based fluorescent ‘AND’ logic gate probes for the detection of homocysteine and a chosen biological analyte†

**DOI:** 10.1039/c9ra04908h

**Published:** 2019-08-27

**Authors:** Luling Wu, Jordan E. Gardiner, Lokesh K. Kumawat, Hai-Hao Han, Ruiying Guo, Xin Li, Xiao-Peng He, Robert B. P. Elmes, Adam C. Sedgwick, Steven D. Bull, Tony D. James

**Affiliations:** Department of Chemistry, University of Bath Bath BA2 7AY UK t.d.james@bath.ac.uk chssdb@bath.ac.uk; Department of Chemistry, University of Texas at Austin 105 E 24th Street A5300 Austin TX 78712-1224 USA a.c.sedgwick@utexas.edu; Department of Chemistry, Maynooth University Human Health Institute, Maynooth University, National University of Ireland Maynooth, County Kildare Ireland Robert.Elmes@mu.ie; Key Laboratory for Advanced Materials, Joint International Research Laboratory of Precision Chemistry and Molecular Engineering, Feringa Nobel Prize Scientist Joint Research Center, School of Chemistry and Molecular Engineering, East China University of Science and Technology 130 Meilong Rd Shanghai 200237 China xphe@ecust.edu.cn; College of Pharmaceutical Sciences, Zhejiang University Hangzhou 310058 China lixin81@zju.edu.cn; Synthesis and Solid State Pharmaceutical Centre, Maynooth University Ireland

## Abstract

With this research we set out to develop a number of coumarin-based ‘AND’ logic fluorescence probes that were capable of detecting a chosen analyte in the presence of HCys. Probe JEG-CAB was constructed by attaching the ONOO^−^ reactive unit, benzyl boronate ester, to a HCys/Cys reactive fluorescent probe, CAH. Similarly, the core unit CAH was functionalised with the nitroreductase (NTR) reactive *p*-nitrobenzyl unit to produce probe JEG-CAN. Both, JEG-CAB and JEG-CAN exhibited a significant fluorescence increase when exposed to either HCys and ONOO^−^ (JEG-CAB) or HCys and NTR (JEG-CAN) thus demonstrating their effectiveness to function as AND logic gates for HCys and a chosen analyte.

## Abstract 2

With this research we set out to develop of a number of coumarin-based ‘AND’ logic fluorescence probes that were capable of detecting a chosen analyte in the presence of HCys.

## Graphical abstract

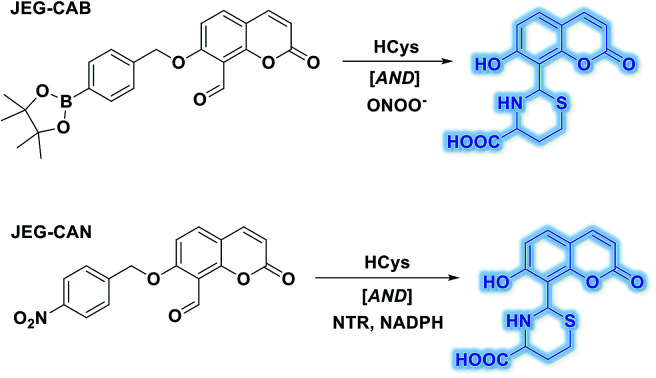

Homocysteine (HCys) is a non-proteinogenic amino acid, formed from the de-methylation of methionine,^1^ which is then converted into cysteine (Cys) *via* a vitamin B_6_ cofactor. Typical physiological concentrations of HCys range between 5–15 μmol L^−1^.^2^ However, elevated levels of HCys (>15 μmol L^−1^), which is known as hyperhomocysteinemia (hHCys),^3^ have been associated with pregnancy disorders, Alzheimer's disease, cardiovascular disease and neurodegenerative diseases (NDs).^4–6^ It is believed that the main cause of HCys induced toxicity is through the non-enzymatic modification of proteins. This is achieved through irreversible covalent attachment of the predominant metabolite of HCys, homocysteine thiolactone (HTL), to lysine residues; a phenomenon known as ‘protein *N*-homocysteinylation’ that results in the loss of a proteins structural integrity leading to loss of enzymatic function and aggregation.^7^

A number of fluorescent sensors have been developed for the detection of HCys to help improve our understanding of its role in biological systems.^8–11^ However, these fluorescent probes have focused on the detection of a single biomarker (HCys), however, processes associated with HCys induced toxicity often involve more than one biochemical species. For example, it has been reported that peroxynitrite (ONOO^−^) and nitric oxide (NO˙) play a significant role in HCys-mediated apoptosis in trigeminal sensory neurons^12^ and HCys has been reported to induce cardiomyocytes cell death through the generation of ONOO^−^.^13^ The production of ONOO^−^ is believed to be the result of an increased production of superoxide (O_2_˙^−^) by HCys activating the enzyme NADPH oxidase.^14–16^ This increased production of O_2_˙^−^ leads to a reduction in the bioavailability of NO˙ by increasing the formation of ONOO^−^ (NO˙ + O_2_˙^−^ → ONOO^−^).^17^ The reported ONOO^−^ concentrations *in vivo* are believed to be approximately 50 μM but, higher concentrations of 500 μM have been found in macrophages.^18,19^ Furthermore, hypoxia has been reported to facilitate HCys production in vitamin-deficient diets^20^ where hypoxia leads to an upregulation of nitroreductase (NTR) – a reductive enzyme upregulated in cells under hypoxic stress.^21,22^ Therefore, the development of tools that enable an understanding of the relationship of HCys with these biologically important species would be highly desirable.

To achieve this, a number of fluorescent probes have been developed that are capable of detecting two or more analytes.^23^ These include AND logic gate based-fluorescence probes, which require both analytes to work in tandem to produce a measurable optical output.^24–28^ In our group, we have developed several ‘AND’ reaction-based probes for the detection of ROS/RNS and a second analyte.^29–32^ These ‘AND’ logic scaffolds have been used to detect two analytes within the same biological system.^24,33^

Owing to the pathological role of HCys, we set out to develop the first example of a fluorescent probe for the detection of HCys and biological related analyte. Aiming towards that target, we became interested in a previously reported coumarin-based fluorescent probe developed by Hong *et al.*CAH, with a salicylaldehyde (Fig. 1).^34^ Salicylaldehyde is a known reactive unit towards HCys/Cys, therefore we believed CAH could be used as a scaffold for the development of ‘AND’-based systems for the detection of HCys/Cys and a second analyte.^34^ In the presence of HCys, CAH exhibited a ‘turn-on’ fluorescence response which is attributed to the nucleophilic nature of the nitrogen and sulfur atoms resulting in thiazine ring formation (Scheme S1, Fig. S1 and S2†).^34–36^

**Fig. 1:**
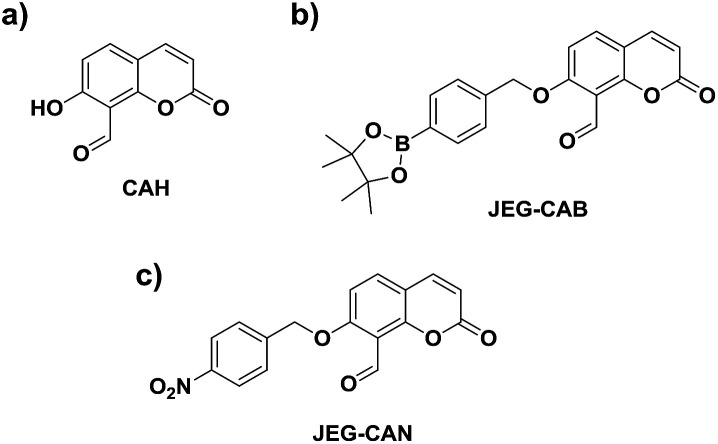
(a) CAH – a core fluorescent unit that enables the synthesis of ‘AND’ based fluorescent probe for the detection of HCys/Cys and a second analyte. (b) JEG-CAB enables the detection of HCys/Cys and (ROS/RNS) while (c) JEG-CAN enables the detection of HCys/Cys and NTR.

We believed that CAH was a useful core unit that can be used to introduce the chosen reactive chemical trigger on the phenol for the detection of the corresponding analyte with HCys/Cys. Owing to the relationship between HCys and ONOO^−^/NTR, we set out on the development of a HCys AND ONOO^−^ probe and a HCys AND NTR probe.

Therefore, we set out to prepare JEG-CAB and JEG-CAN, which are able to detect HCys/Cys and peroxynitrite (ONOO^−^) or nitroreductase (NTR), respectively (Scheme 1). For JEG-CAB, a benzyl boronate ester was introduced as a ONOO^−^ reactive unit.^37^ For JEG-CAN, a *p*-nitrobenzyl group was installed as it is known to be an effective substrate for NTR.^38–40^

**Scheme 1:**
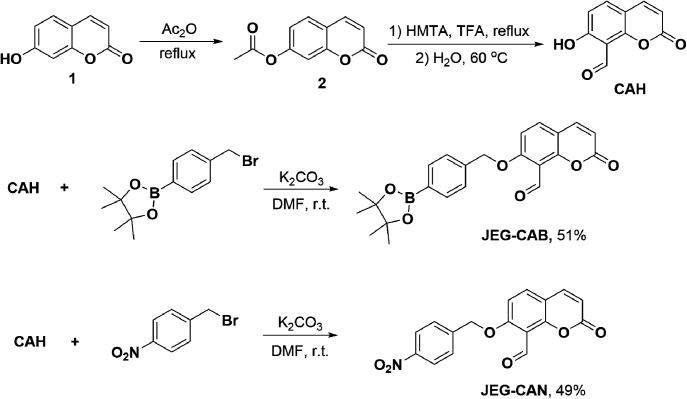
Synthesis of target probe JEG-CAB and JEG-CAN.

To afford CAH, compound 2 was synthesized by refluxing umbelliferone and acetic anhydride at 140 °C. Compound 2 was then dissolved in trifluoroacetic acid at 0 °C followed by the addition of hexamethylenetetramine (HMTA). The mixture was heated to reflux overnight and the solvent was then removed. The intermediate was then hydrolyzed in H_2_O for 30 min at 60 °C. Upon isolating CAH, it was then alkylated using 4-bromomethylphenylboronic acid pinacol ester and K_2_CO_3_ in DMF at r.t. to afford JEG-CAB in 51% yield. JEG-CAN was produced by alkylating CAH using 4-nitrobenzyl bromide and K_2_CO_3_ in DMF at r.t. to give 49% yield (Scheme 1).

We then evaluated the ability of JEG-CAB to detect ONOO^−^ ‘AND’ HCys in PBS buffer solution (10 mM, pH 7.40). The maximum absorption of JEG-CAB at 336 nm shifted to 323 nm with the addition of HCys and then slightly shifted to 328 nm following the addition of ONOO^−^ (Fig. S3†). As shown in Fig. 2, JEG-CAB was initially non-fluorescent, but the addition of HCys (1 mM) led to a small increase in fluorescence intensity, the subsequent additions of ONOO^−^ (0–24 μM) led to a significant increase in fluorescence intensity (>17-fold, see Fig. S5†). These results demonstrated the requirement for both ONOO^−^ ‘AND’ HCys to obtain a significant turn ‘‘on’’ fluorescence response.

**Fig. 2:**
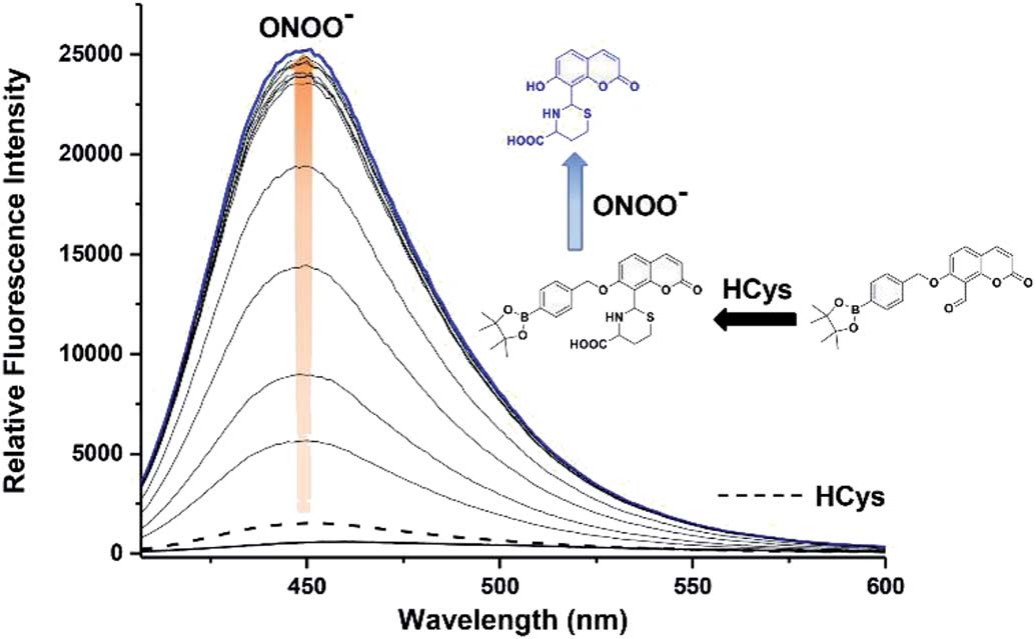
Fluorescence spectra of JEG-CAB (15 μM) with addition of HCys (1 mM) and incubated for 40 min then measured. Followed by incremental additions of ONOO^−^ (0–24 μM). The data was obtained in PBS buffer solution (pH 7.40, 10 mM) at 25 °C. *λ*_ex_ = 371 (bandwidth 20) nm. Dashed line represents JEG-CAB and Hcys addition only. Blue line represents highest intensity after addition of ONOO^−^.

The addition of HCys and ONOO^−^ were then performed in reverse where JEG-CAB exhibited a negligible increase in fluorescence intensity upon addition of ONOO^−^ (16 μM). However, in a similar manner to that shown in Fig. 2, a large increase in fluorescence intensity was produced after the subsequent addition of HCys (0–5.5 mM) (Fig. 3 and S6†). LC-MS experiments were carried out to ascertain the reaction mechanism and the results confirmed the sequential formation of the thiazine ring in the presence of HCys followed by boronate ester cleavage in the presence of ONOO^−^ or *vice versa* (Scheme S2 and Fig. S19–S21†).

**Fig. 3:**
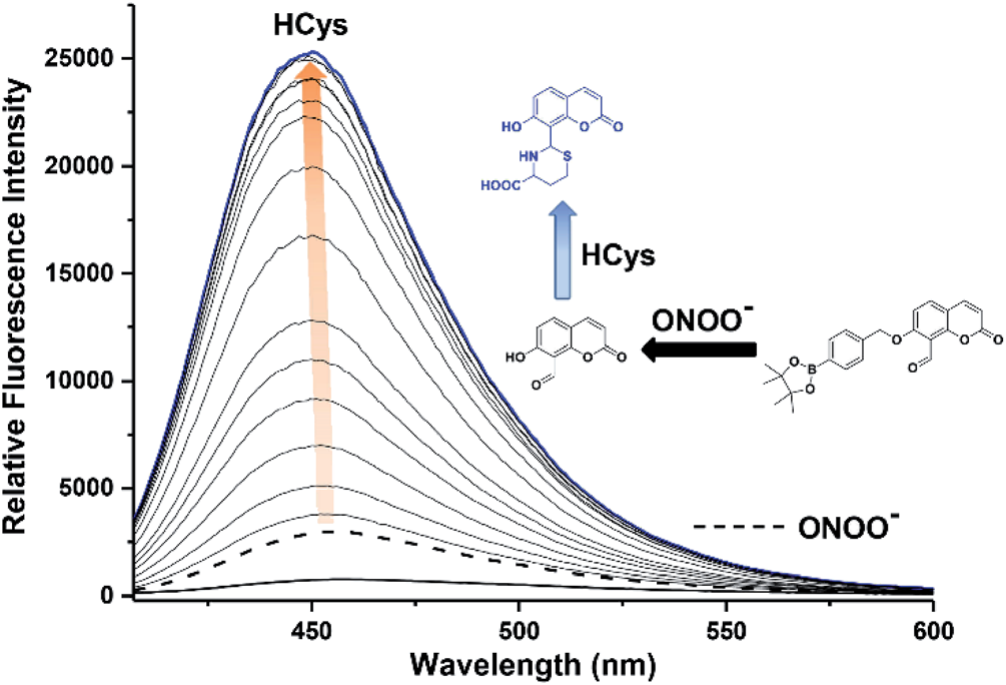
Fluorescence spectra of JEG-CAB (15 μM) with addition of ONOO^−^ (16 μM) and followed by incremental additions of HCys (0–5.5 mM) measurements were taken after 40 min of both additions. The data was obtained in PBS buffer solution (pH 7.40, 10 mM) at 25 °C. *λ*_ex_ = 371 (bandwidth 20) nm. Dashed line represents JEG-CAB and ONOO^−^ addition only. Blue line represents highest intensity after addition of HCys.

As expected, probe JEG-CAB was shown to have excellent selectivity with ONOO^−^ against other ROS in the presence of HCys (1 mM) (Fig. S9 and S10†). Furthermore, JEG-CAB exhibited a high degree of selectivity towards a series of amino acids where only HCys and Cys led to a fluorescence response in the presence of ONOO^−^. This is due to the formation of stable five or six-membered thiazine rings (Fig. S7 and S8†).^34^

We then evaluated the changes in the fluorescence of JEG-CAN with both HCys and NTR in PBS buffer solution (10 mM, pH 7.40, containing 1% DMSO). As shown in Fig. 4, addition of HCys led to a small increase in fluorescence intensity. However, subsequent addition of NTR (4 μg mL^−1^) led to a large time dependant increase in fluorescence intensity. To ensure both analytes were required, NTR and NADPH was kept constant (4 μg mL^−1^ and 400 μM respectively) resulting in a 3.4 fold fluorescence increase (Fig. 5). We attribute the large initial increase to background fluorescence of NADPH.^41^ NTR then facilitates reduction of the nitro group of JEG-CAN releasing the core probe CAH*via* a fragmentation cascade (Scheme S3†).^38,42^ Subsequent addition of HCys (2.0 mM) led to a 2 fold increase in fluorescence intensity. Again, LC-MS experiments confirmed the proposed reaction mechanism (Fig. S22†).

**Fig. 4:**
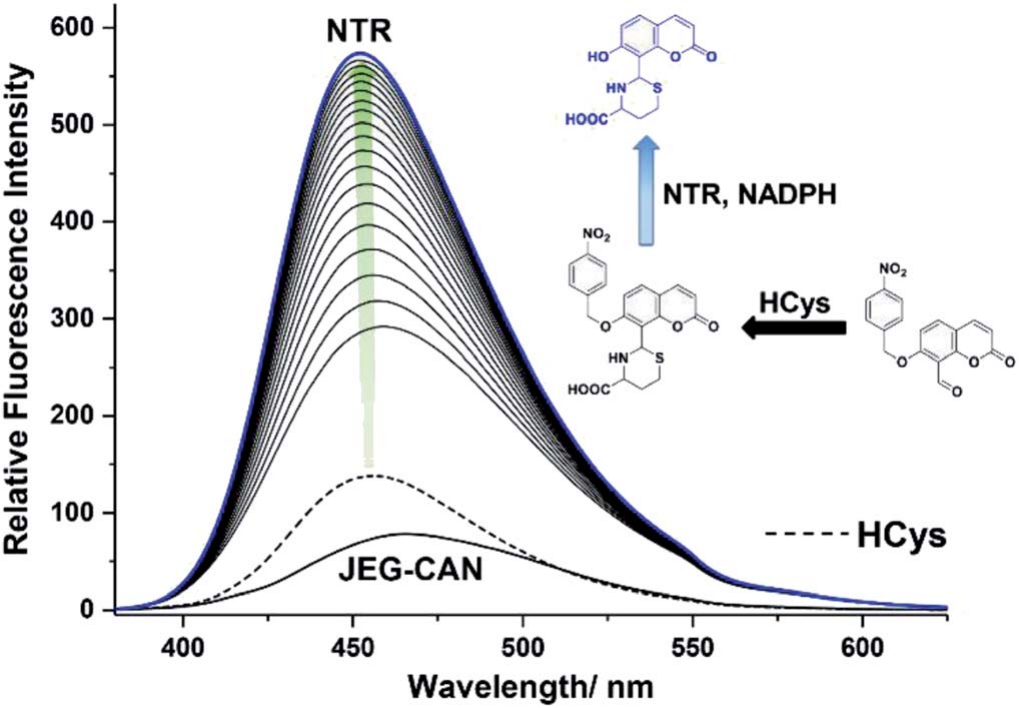
Fluorescence spectra of JEG-CAN (15 μM) with initial addition of HCys (2 mM) and incubated for 60 min. Followed by addition of nitroreductase (4 μg mL^−1^) and NADPH (400 μM) and measured over 90 min in PBS buffer solution (pH = 7.40, 10 mM, containing 1% DMSO). *λ*_ex_ = 363 nm. Ex slit: 5 nm and em slit: 5 nm. Dashed line represents JEG-CAN and HCys addition only. Blue line represents highest intensity after addition of NTR.

**Fig. 5:**
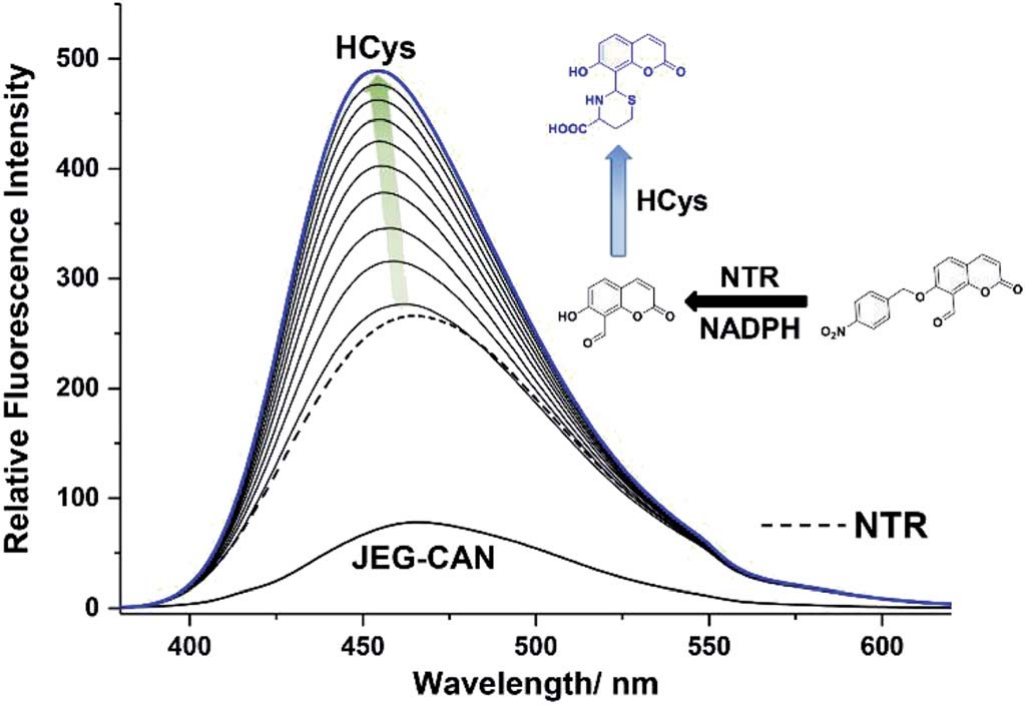
Fluorescence spectra of JEG-CAN (15 μM) with initial addition of nitroreductase (4 μg mL^−1^) and NADPH (400 μM) and incubated for 60 min. Followed by addition HCys (2 mM) and measured over 90 min in PBS buffer solution (pH = 7.40, 10 mM, containing 1% DMSO). *λ*_ex_ = 363 nm. Ex slit: 5 nm and em slit: 5 nm. Dashed line represents JEG-CAN and NTR addition only. Blue line represents highest intensity after addition of HCys.

Kinetic studies for JEG-CAN with both NTR and HCys were carried out (Fig. S11–S18†) where it is clear that JEG-CAN exhibits a dose dependant fluorescence increase in response of both HCys and NTR.

Unfortunately, the probes failed to give good data in cells, which could be due to their short excitation wavelengths or the extremely low intracellular HCys concentrations (5–15 μM). We are now pursuing the development ‘AND’ logic fluorescence probes with longer excitation and emission wavelengths suitable for *in vitro* and *in vivo* applications.

In summary, we have developed two coumarin-based ‘AND’ logic fluorescence probes (JEG-CAB and JEG-CAN) for the detection of HCys/Cys and ONOO^−^ or NTR, respectively. CAH is a useful platform that enables easy modification for the development of ‘AND’-based fluorescent probes for the detection of HCys/Cys and a second analyte. Both JEG-CAB and JEG-CAN were shown to be ‘AND’-based fluorescent probes.

## Conflicts of interest

No conflicts of interest.

## Supplementary Material

RA-009-C9RA04908H-s001
