# Supplementary material for: Coumarin-based fluorescent ‘AND’ logic gate probes for the detection of homocysteine and a chosen biological analyte
Source: RSC Adv. 2019 Aug 27;9(45):26425–8. doi: 10.1039/c9ra04908h (PMC9070123; doi:10.1039/c9ra04908h)
Supplement: RA-009-C9RA04908H-s001 [file RA-009-C9RA04908H-s001.pdf]

Electronic Supplementary Material (ESI) for RSC Advances.
This journal is © The Royal Society of Chemistry 2019

# Coumarin-based fluorescent 'AND' logic probes for the detection of homocysteine and a chosen biological analyte

Luling Wu,[a] Jordan E. Gardiner,[a] Lokesh K. Kumawat,[c] Hai-Hao Han,[d] Ruiying Guo,[e] Xin Li,*[e] Xiao-Peng He,*[d] Robert B. P. Elmes,*[cf] Adam C. Sedgwick,*[b] Steven D. Bull,*[a] and Tony D. James*[a]

[a]Department of Chemistry, University of Bath, Bath, BA2 7AY, UK.

[b]Department of Chemistry. University of Texas at Austin, 105 E 24th street A5300, Austin, TX 78712-1224, United States.

[c]Department of Chemistry and Maynooth University Human Health Institute, Maynooth University, National University of Ireland, Maynooth, County Kildare, Ireland.

[d]Key Laboratory for Advanced Materials and Joint International Research Laboratory of Precision Chemistry and Molecular Engineering, Feringa Nobel Prize Scientist Joint Research Center, School of Chemistry and Molecular Engineering, East China University of Science and Technology, 130 Meilong Rd., Shanghai 200237, China.

[e]College of Pharmaceutical Sciences, Zhejiang University, Hangzhou 310058, China.

[f]Synthesis and Solid State Pharmaceutical Centre, Maynooth University, Ireland.

**Email:** t.d.james@bath.ac.uk, chssdb@bath.ac.uk, a.c.sedgwick@utexas.edu, robert.elmes@mu.ie, xphe@ecust.edu.cn, lixin81@zju.edu.cn

**Table of Contents**


**1. Reaction mechanisms** S3

**2. Generation of various ROS** S4

**3. Fluorescence analysis of CAH.** S5

**4. UV-Vis and fluorescence analysis of JEG-CAB** S6-9

**5. Fluorescence analysis of JEG-CAN** S10-17

**6. Mass spec analysis of JEG-CAB** S18-20

**7. Mass spec analysis of JEG-CAN** S21

**8. Experimental** S22-23

**9. NMR spectrum** S24-31

**10. References** S32

**11. Author Contributions** S32

# 1. Reaction mechanisms

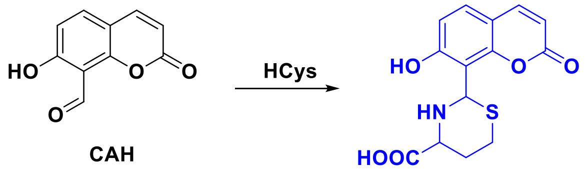


**Scheme S1.** Reaction mechanism of **CAH** with the addition of homocysteine (HCys).[1]

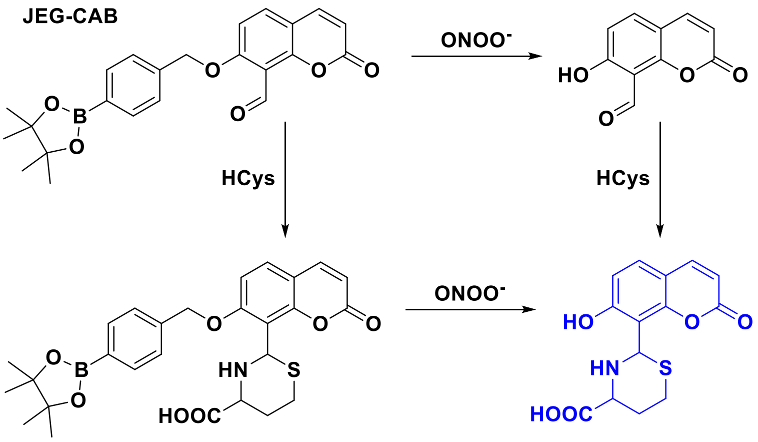


**Scheme S2.** Reaction of **JEG-CAB** with HCys and peroxynitrite ($ONOO^-$).

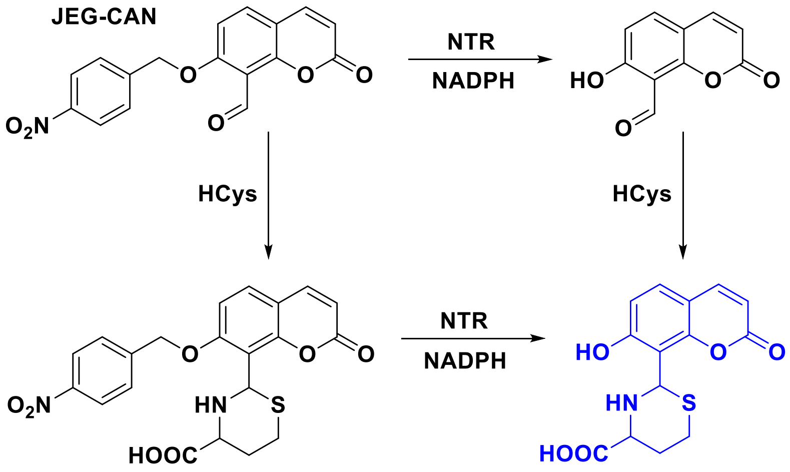


**Scheme S3.** Reaction of **JEG-CAN** with HCys and nitroreductase (NTR) and NADPH.

## 2. Generation of various ROS

**ROO•**

ROO• was generated from 2,2'-azobis (2-amidinopropane) dihydrochloride. AAPH (2, 2' azobis (2-amidinopropane) dihydrochloride, 1 M) was added into deionizer water, and then stirred at 37 °C for 30 min.

**$O_2^{\bullet-}$**

Superoxide was generated from $KO_2$. $KO_2$ and 18-crown-6 ether (2.5 eq) was dissolved in DMSO to afford a 0.25 M solution.

**•OH**

Hydroxyl radical was generated by the Fenton reaction. To prepare •OH solution, hydrogen peroxide ($H_2O_2$, 10 eq) was added to $Fe(ClO_4)_2$ in deionised water.

**$^1O_2$**

$^1O_2$ was generated by reacting $H_2O_2$ (1 mM) with NaClO (1 mM). The solution of $H_2O_2$ was added in one portion to the aqueous solution of NaClO and stir for 2 minutes, using the prepared solution immediately.

**$ONOO^-$**

Simultaneously, 0.6 M $KNO_2$, 0.6 M in HC1, 0.7 M in $H_2O_2$ was added at to a 3 M NaOH solution at 0 ºC. The concentration of peroxynitrite was estimated by using extinction co-efficient of 1670 $M^{-1}$ $cm^{-1}$ at 302 nm in 0.5 M sodium hydroxide aqueous solutions.

**$ClO^-$**

The concentration of $ClO^-$ was determined from the absorption at 292 nm ($\varepsilon$ = 350 $M^{-1}$ $cm^{-1}$).

**$H_2O_2$**

The concentration of $H_2O_2$ was determined from the absorption at 240 nm ($\varepsilon$ = 43.6 $M^{-1}$ $cm^{-1}$).

## 3. Fluorescence analysis of CAH

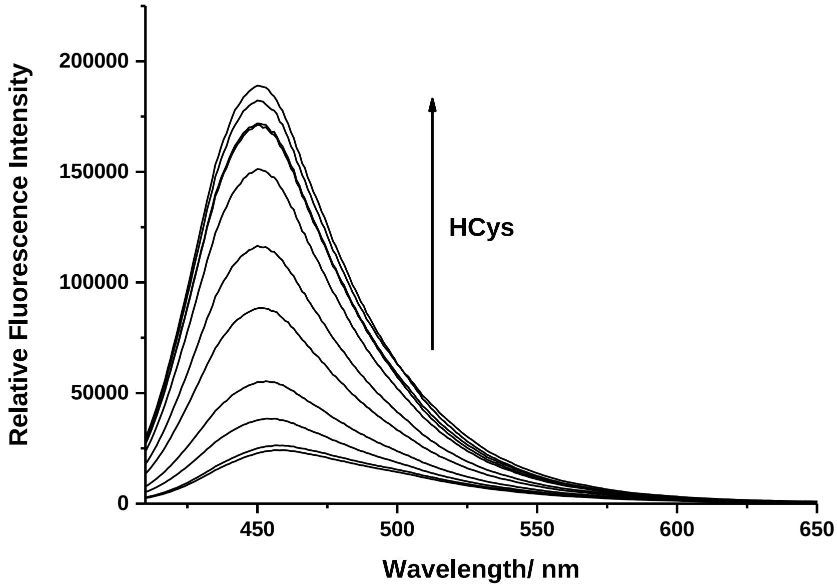


**Figure S1.** Fluorescence spectra of **CAH** (15 μM) with in increasing addition of HCys (from 0 to 5000 μM) PBS buffer (pH = 7.40) after 40 min. $\lambda_{ex}$ = 353 (bandwidth 15) nm.

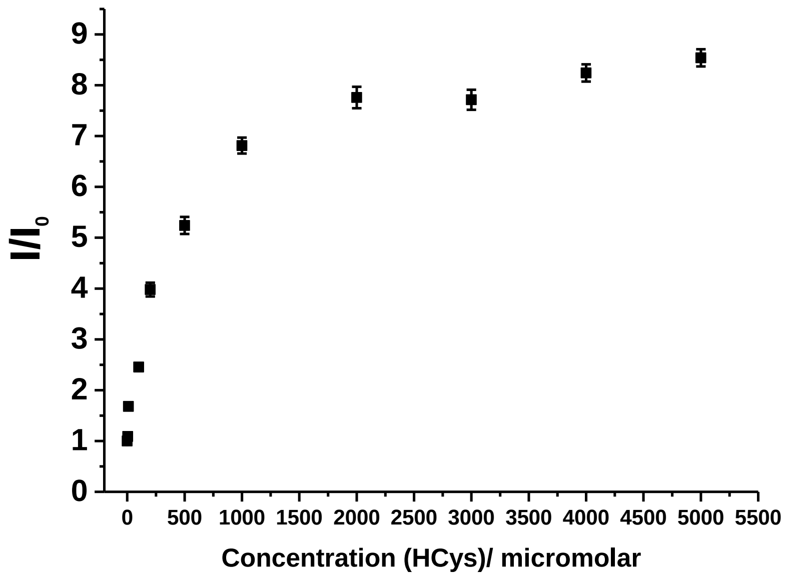


**Figure S2.** Fluorescence intensity changes ($I/I_0$) of probe **CAH** (15 μM) with addition of HCys (0 – 5000 μM). 40 min wait between addition in PBS buffer solution (pH = 7.40). $\lambda_{ex}$ = 353 (bandwidth 15 nm)/ $\lambda_{em}$ = 448 nm.

## 4. UV-Vis and fluorescence analysis of JEG-CAB

Fluorescence measurements of **JEG-CAB** were performed on a BMG Labtech CLARIOstar using Greiner Bio-One microplates (96-well, PS, f-bottom (chimney well), black-walled). Data were collected via the BMG Labtech Clariostar data analysis software package MARS.

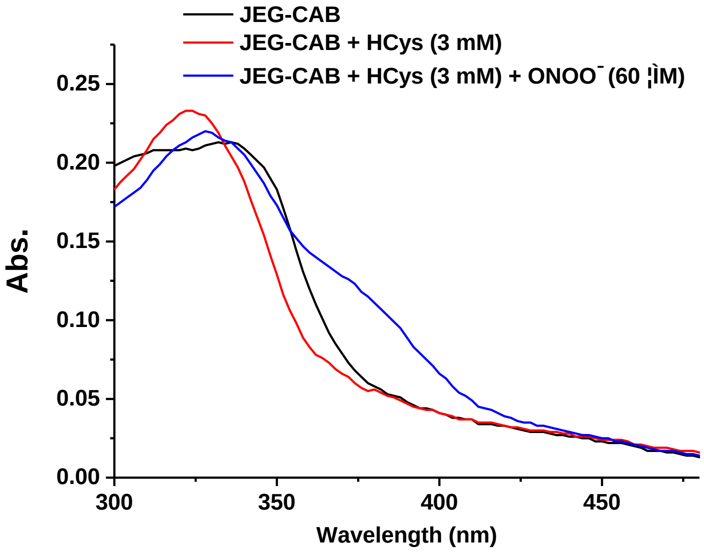


**Figure S3.** Absorption spectrum of **JEG-CAB** (30 µM) with and without HCys (3 mM), and **JEG-CAB** (30 µM) with addition of HCys (3 mM) wait 40 min then addition of $ONOO^-$ (60 µM) in PBS buffer solution (pH 7.40 at 25 °C).

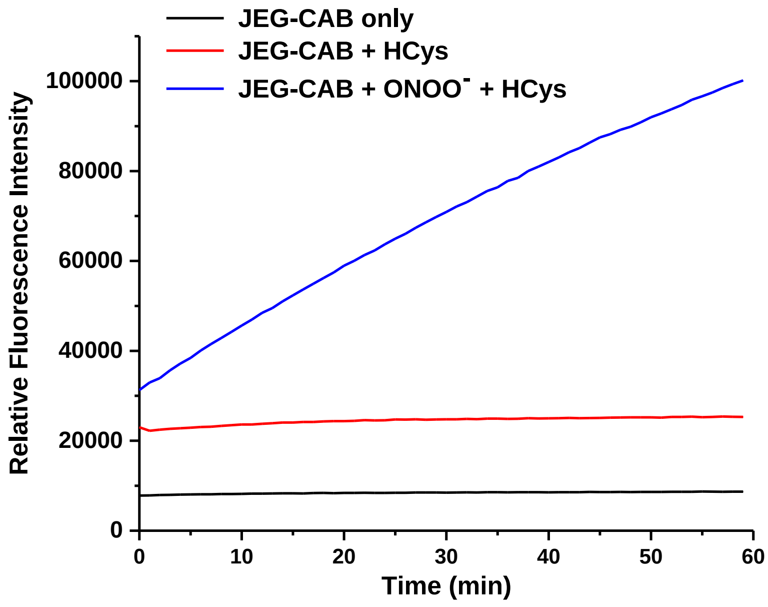


**Figure S4.** Fluorescence intensity changes over time of **JEG-CAB** (15 µM). Red line - addition of HCys only (1 mM). Blue line - addition of $ONOO^-$ (18 µM) followed by HCys (1 mM). All experiments were carried out in PBS buffer solution (pH 7.40). $\lambda_{ex}$ = 371 (bandwidth 20) nm/ $\lambda_{em}$ = 448 nm.

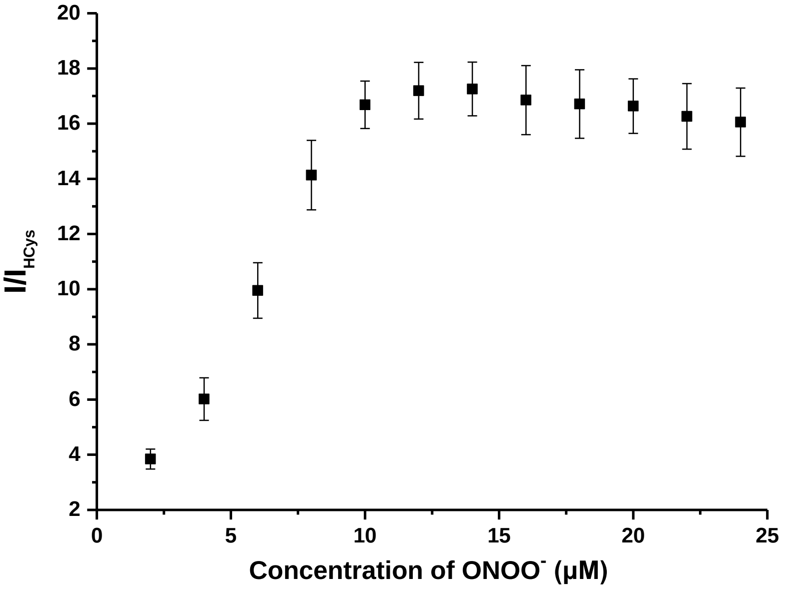


**Figure S5.** Fluorescence intensity changes ($I/I_{HCys}$) for **JEG-CAB** (15 μM) with addition of HCys (1 mM) wait 40 min then additions of $ONOO^-$ (0 - 24 μM) in PBS buffer solution (pH = 7.40, 10 mM). Fluorescence intensities were measured with $\lambda_{ex}$ = 371 (bandwidth 20) nm/$\lambda_{em}$ = 448 nm.

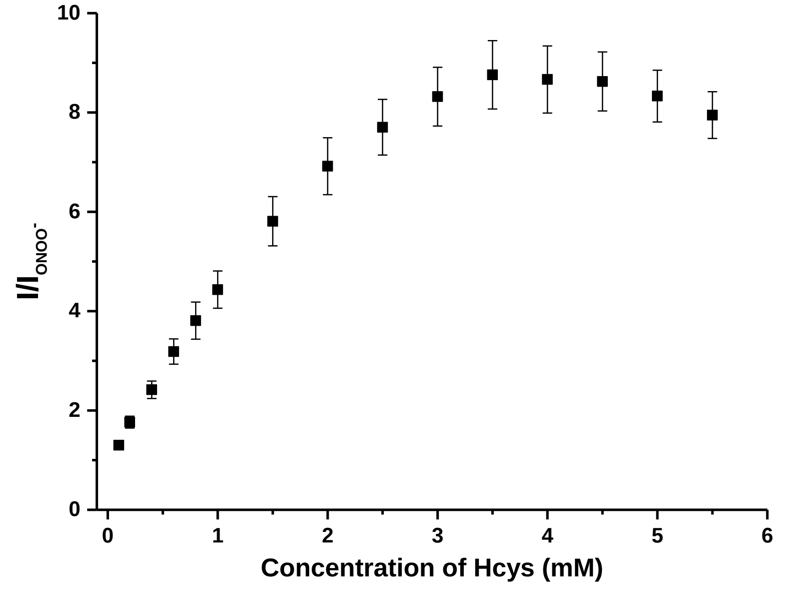


**Figure S6.** Fluorescence intensity changes ($I/I_{ONOO^-}$) for **JEG-CAB** (15 μM) with addition of $ONOO^-$ (16 μM) wait 1 min then additions of HCys (0 - 5.5 mM) with 40 min incubation before measurement in PBS buffer solution (pH = 7.40). Fluorescence intensities were measured with $\lambda_{ex}$ = 371 (bandwidth 20) nm/$\lambda_{em}$ = 448 nm.

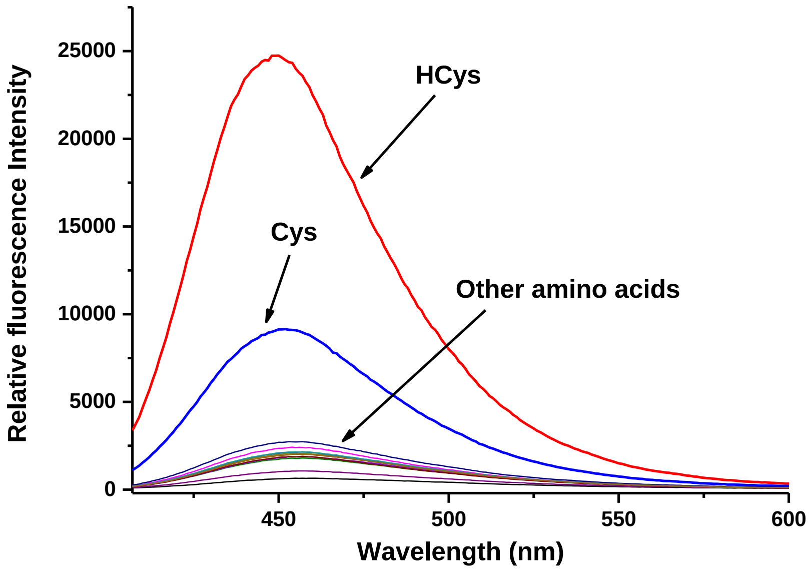


**Figure S7.** Changes in fluorescence emission of **JEG-CAB** (15 μM) with initial addition of $ONOO^-$ (16 μM) then addition of various amino acids (2.5 mM) after 40 min in PBS buffer solution (pH = 7.40). $\lambda_{ex}$ = 371 (bandwidth 20) nm.

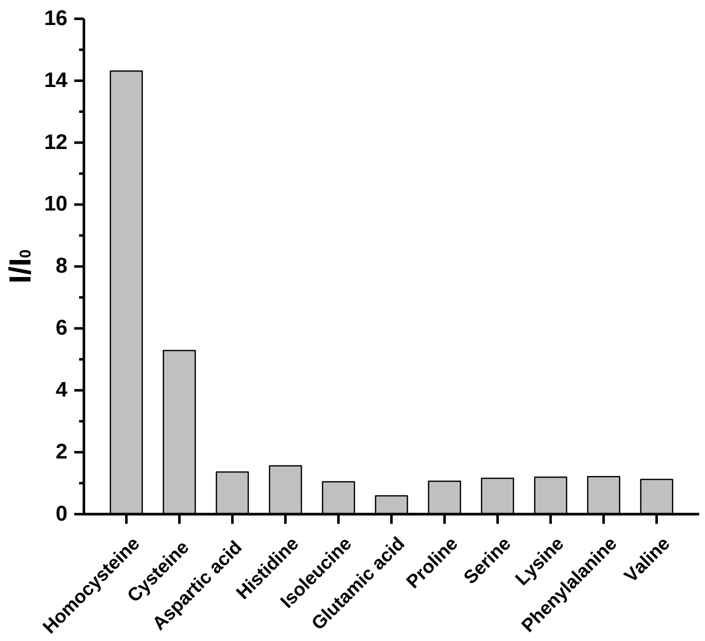


**Figure S8.** Selectivity bar chart of **JEG-CAB** (15 μM) with addition of $ONOO^-$ (16 μM) then addition of various amino acids (2.5 mM). 40 min wait before measurement in PBS buffer solution (pH = 7.40). Fluorescence intensities were measured with $\lambda_{ex}$ = 371 (bandwidth 20) nm/ $\lambda_{em}$ = 448 nm.

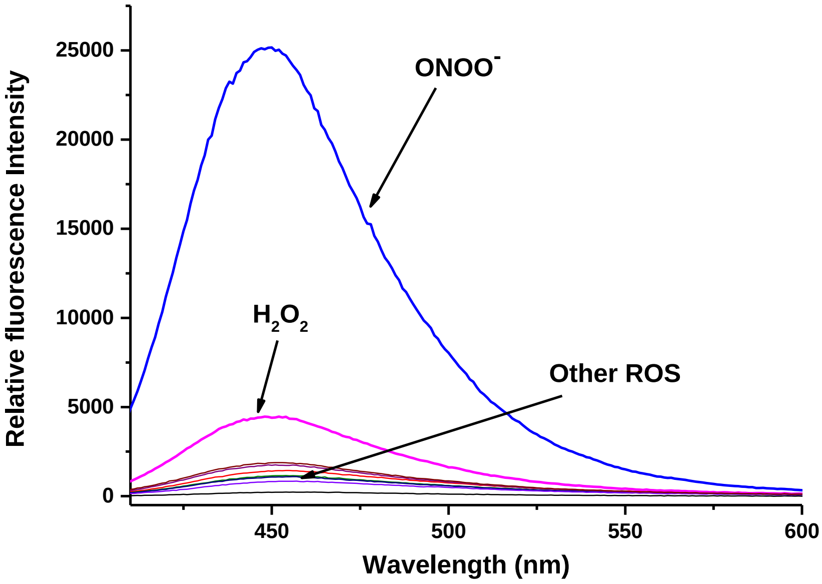


**Figure S9.** Changes in fluorescence emission of **JEG-CAB** (15 µM) with initial addition of HCys (1 mM) wait 40 min then addition of $ONOO^-$ (10 µM) wait 1 min and various other ROS (100 µM) after 30 min in PBS buffer solution (pH = 7.40). $\lambda_{ex}$ = 371 (bandwidth 20) nm.

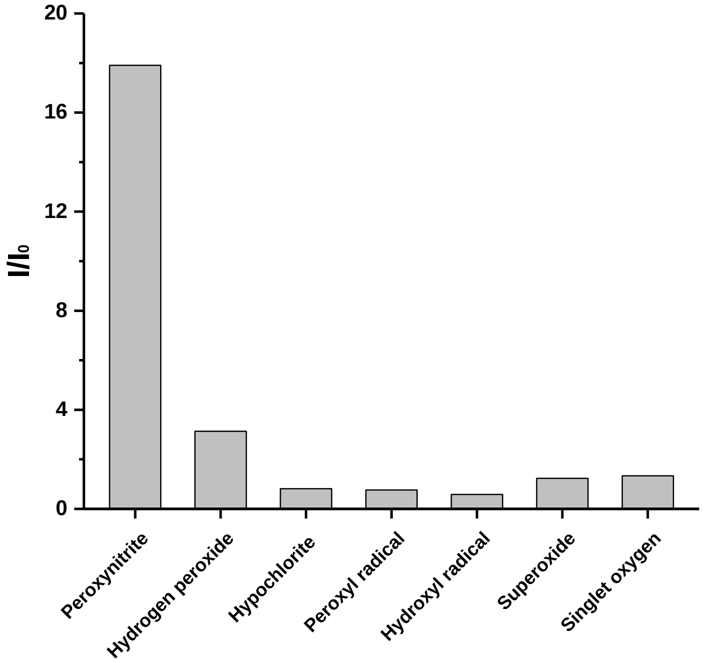


**Figure S10.** Selectivity bar chart of **JEG-CAB** (15 µM) with addition of HCys (1 mM) wait 40 min then addition of $ONOO^-$ (10 µM) wait 1 min and various other ROS (100 µM) wait 30 min before measurement in PBS buffer solution (pH = 7.40). Fluorescence intensities were measured with $\lambda_{ex}$ = 371 (bandwidth 20) nm/ $\lambda_{em}$ = 448 nm.

## 5. Fluorescence analysis of JEG-CAN

Fluorescence titrations of **JEG-CAN** were carried out using a Jasco FP-6300 spectrofluorometer with slit width ex. 5.0 nm and em. 5.0 nm in PBS buffer (pH 7.40, containing 1% DMSO).

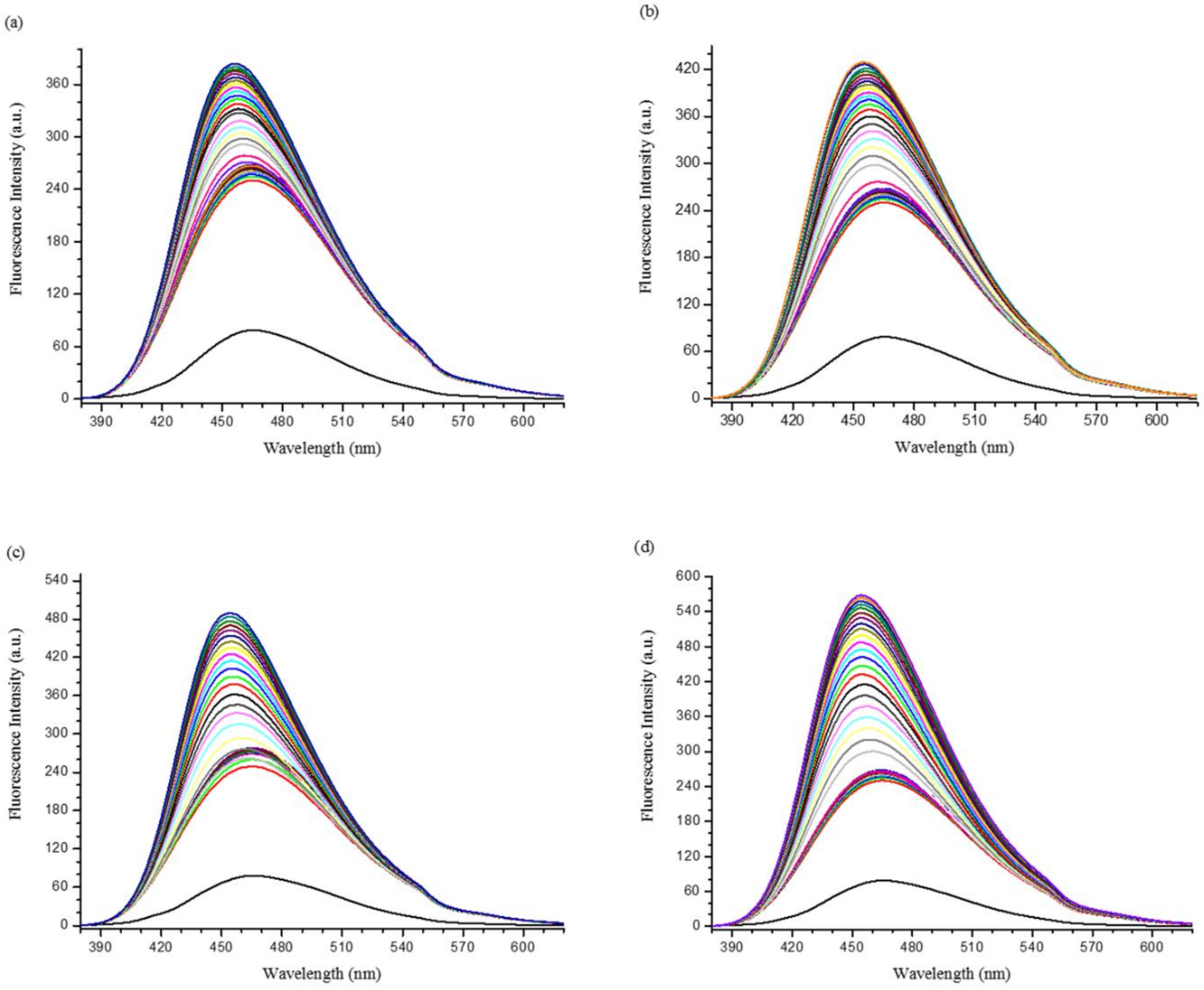


**Figure S11.** Fluorescence spectra of **JEG-CAN** (15 µM) with initial addition of NADPH (400 µM) and NTR (4 µg/mL) incubated for 90 min, followed by the addition of HCys ((a) 0.5 mM, (b) 1.0 mM, (c) 2.0 mM, (d) 4.0 mM) and monitored for a further 120 minutes. Fluorescence intensities were measured in PBS buffer (pH 7.40, containing 1% DMSO) with $\lambda_{ex}$ = 363 nm. Ex slit: 5 nm and em slit: 5 nm

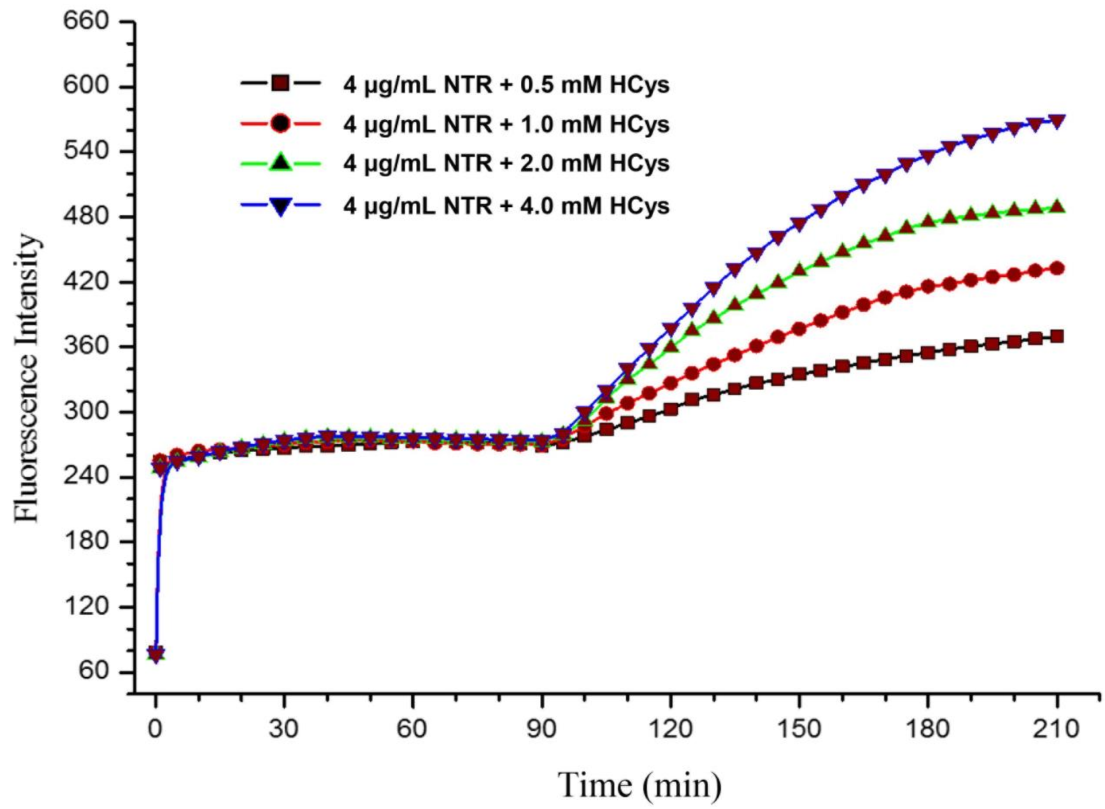


**Figure S12.** A plot of the fluorescence intensity changes at 453 nm depending on time. **JEG-CAN** (15 μM) with initial addition of NADPH (400 μM) and NTR (4 μg/mL) and incubated for 90 min, followed by the addition of HCys (0.5 mM, 1.0 mM, 2.0 mM, 4.0 mM respectively) and monitored for a further 120 minutes. Fluorescence intensities were measured in PBS buffer (pH 7.40, containing 1% DMSO) with $\lambda_{ex}$ = 363 nm. Ex slit: 5 nm and em slit: 5 nm.

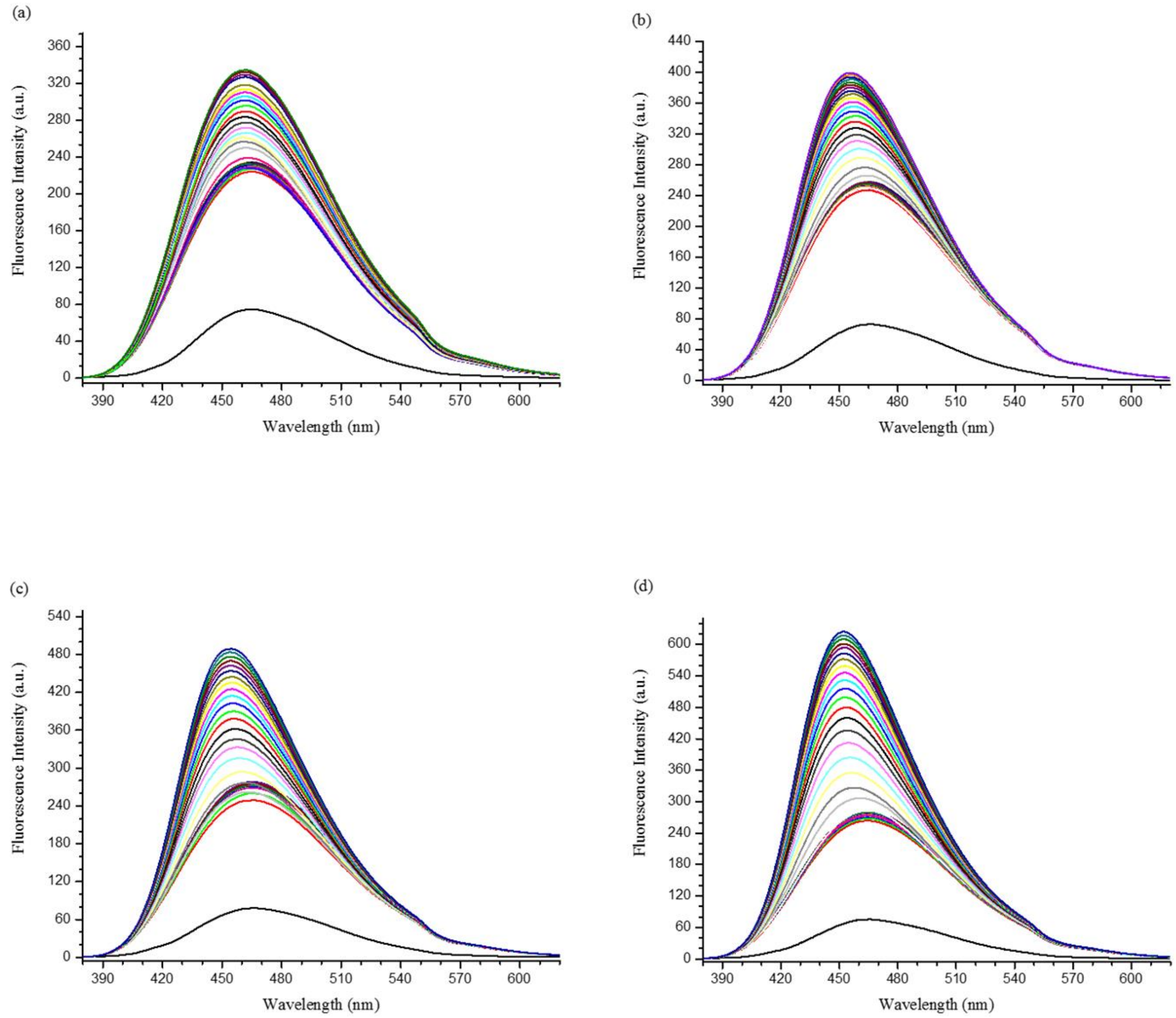


**Figure S13.** Fluorescence spectra of **JEG-CAN** (15 μM) with initial addition of NADPH (400 μM) and NTR ((a) 1 μg/mL, (b) 2 μg/mL, (c) 4 μg/mL, (d) 10 μg/mL) and incubated for 90 minutes, followed by the addition of Hcys (2.0 mM) and monitored for a further 120 minutes. Fluorescence intensities were measured in PBS buffer (pH 7.40, containing 1% DMSO) with $\lambda_{ex}$ = 363 nm. Ex slit: 5 nm and em slit: 5 nm.

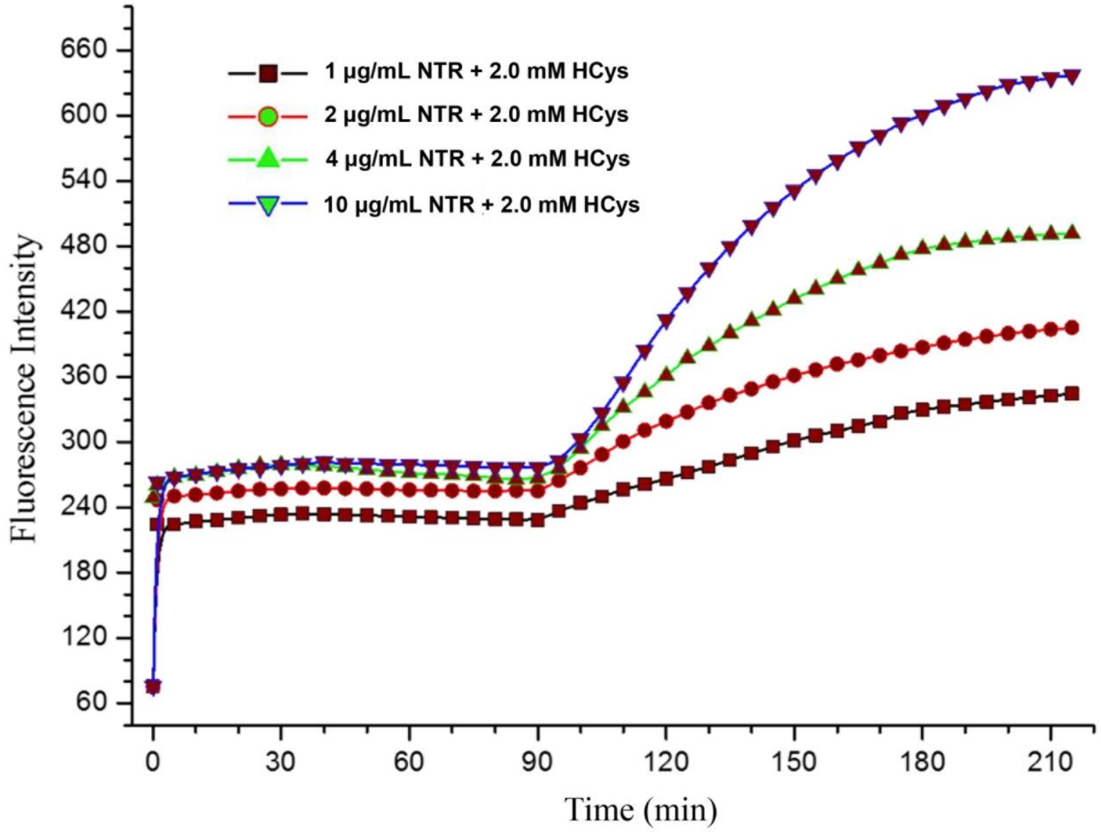


**Figure S14.** A plot of the fluorescence intensity changes at 453 nm depending on time. **JEG-CAN** (15 μM) with initial addition of NADPH (400 μM) and NTR (1 μg/ mL, 2 μg/mL, 4 μg/mL, 10 μg/mL respectively) and incubated for 90 minutes, followed by the addition of Hcys (2.0 mM) and monitored for a further 120 minutes. Fluorescence intensities were measured in PBS buffer (pH 7.40, containing 1% DMSO) with $\lambda_{ex}$ = 363 nm. Ex slit: 5 nm and em slit: 5 nm.

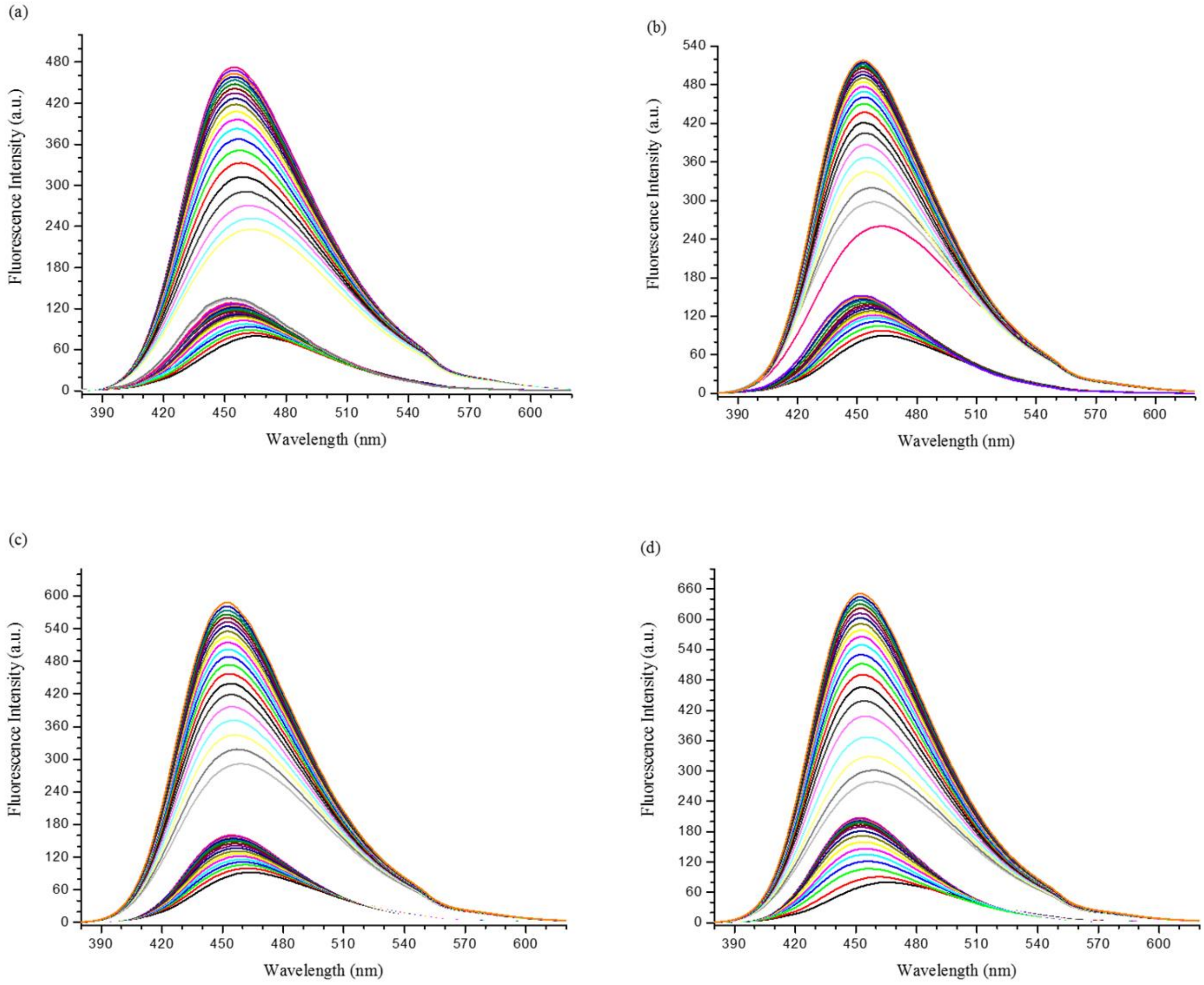


**Figure S15.** Fluorescence spectra of **JEG-CAN** (15 μM) with initial addition of Hcys ((a) 0.5 mM, (b) 1.0 mM, (c) 2.0 mM, (d) 4.0 mM) and incubated for 90 minutes, followed by addition of NADPH (400 μM) and NTR (4 μg/mL) and monitored for a further 120 minutes. Fluorescence intensities were measured in PBS buffer (pH 7.40, containing 1% DMSO) with $\lambda_{ex}$ = 363 nm. Ex slit: 5 nm and em slit: 5 nm.

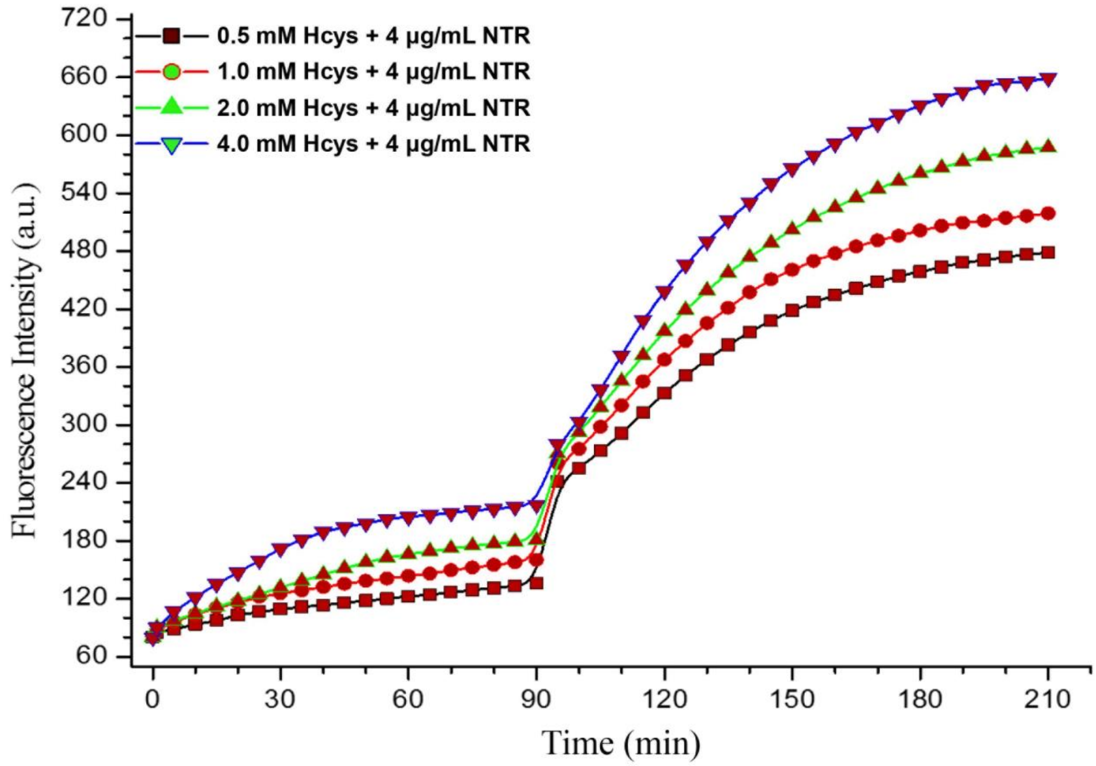


**Figure S16.** A plot of the fluorescence intensity changes at 453 nm depending on time. **JEG-CAN** (15 µM) with initial addition of HCys (0.5 mM, 1.0 mM, 2.0 mM, 4.0 mM respectively) and incubated for 90 min, followed by the addition of NADPH (400 µM) and NTR (4 µg/mL) and monitored for a further 120 minutes. Fluorescence intensities were measured in PBS buffer (pH 7.40, containing 1% DMSO) with $\lambda_{ex}$ = 363 nm. Ex slit: 5 nm and em slit: 5 nm.

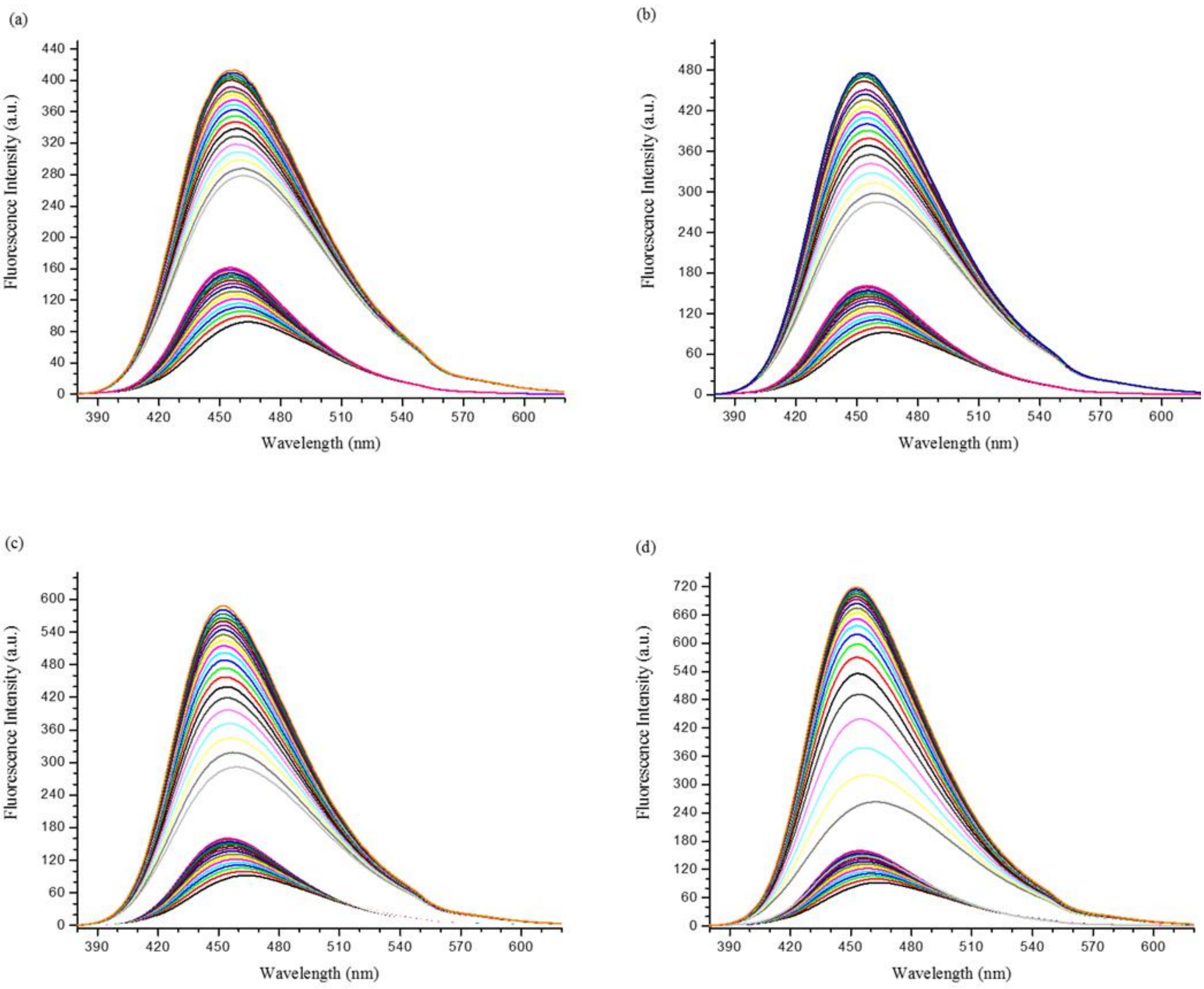


**Figure S17**. Fluorescence spectra of **JEG-CAN** (15 μM) with initial addition of Hcys (2 mM) and incubated for 90 min, followed by addition of NADPH (400 μM) and NTR ((a) 1 μg/mL (b) 2 μg/mL, (c) 4 μg/mL, (d) 10 μg/mL) and incubated for a further 120 minutes. Fluorescence intensities were measured in PBS buffer (pH 7.40, containing 1% DMSO) with $\lambda_{ex}$ = 363 nm. Ex slit: 5 nm and em slit: 5 nm.

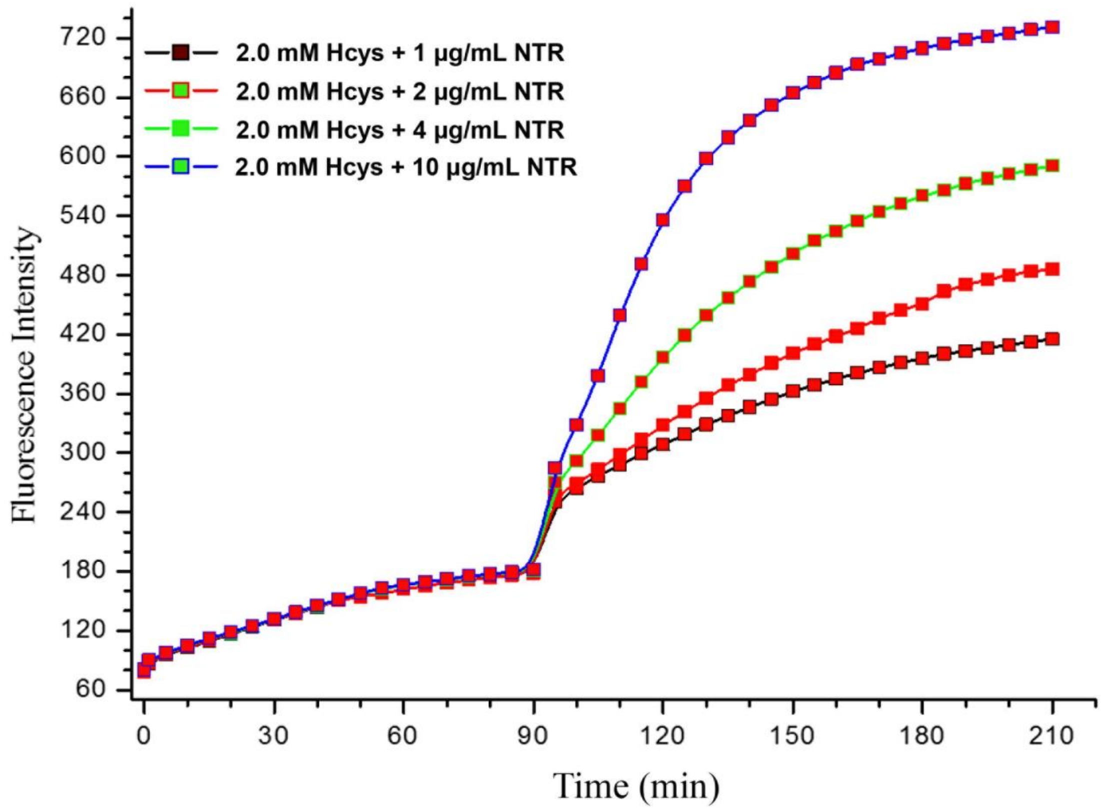


**Figure S18.** A plot of the fluorescence intensity changes at 453 nm depending on time. **JEG-CAN** (15 µM) with initial addition of HCys (2.0 mM) and incubated for 90 min, followed by the addition of NADPH (400 µM) and NTR (1 µg/mL, 2 µg/mL, 4 µg/mL, 10 µg/mL respectively) and monitored for a further 120 minutes. Fluorescence intensities were measured in PBS buffer (pH 7.4, containing 1% DMSO) with $\lambda_{ex}$ = 363 nm. Ex slit: 5 nm and em slit: 5 nm.

## 6. Mass spec analysis of JEG-CAB

LC-MS analyses were performed using an Agilent QTOF 6545 with Jetstream ESI spray source coupled to an Agilent 1260 Infinity II Quat pump HPLC with 1260 autosampler, column oven compartment and variable wavelength detector (VWD). LC-MS experiments were performed in order to determine the detection mechanism for the dual selective sensing strategy with **JEG-CAB** (10 μM) in the presence of $ONOO^-$ (18 μM) and homocysteine (1.0 mM) in water (containing 5% DMSO). Experiments were performed after 1 min of incubation with $ONOO^-$ and 40 min with HCys.

**Compound Table**

| Compound Label | RT (min) | Observed mass (m/z) | Neutral observed mass (Da) | Theoretical mass (Da) | Mass error (ppm) | Isotope match score (%) |
|---|---|---|---|---|---|---|
| Cpd 1: C23 H23 B O6 | 0.95 | 429.1486 | 405.1628 | 405.1624 | 0.93 | 98.75 |

Mass errors of between -5.00 and 5.00 ppm with isotope match scores above 60% are considered confirmation of molecular formulae

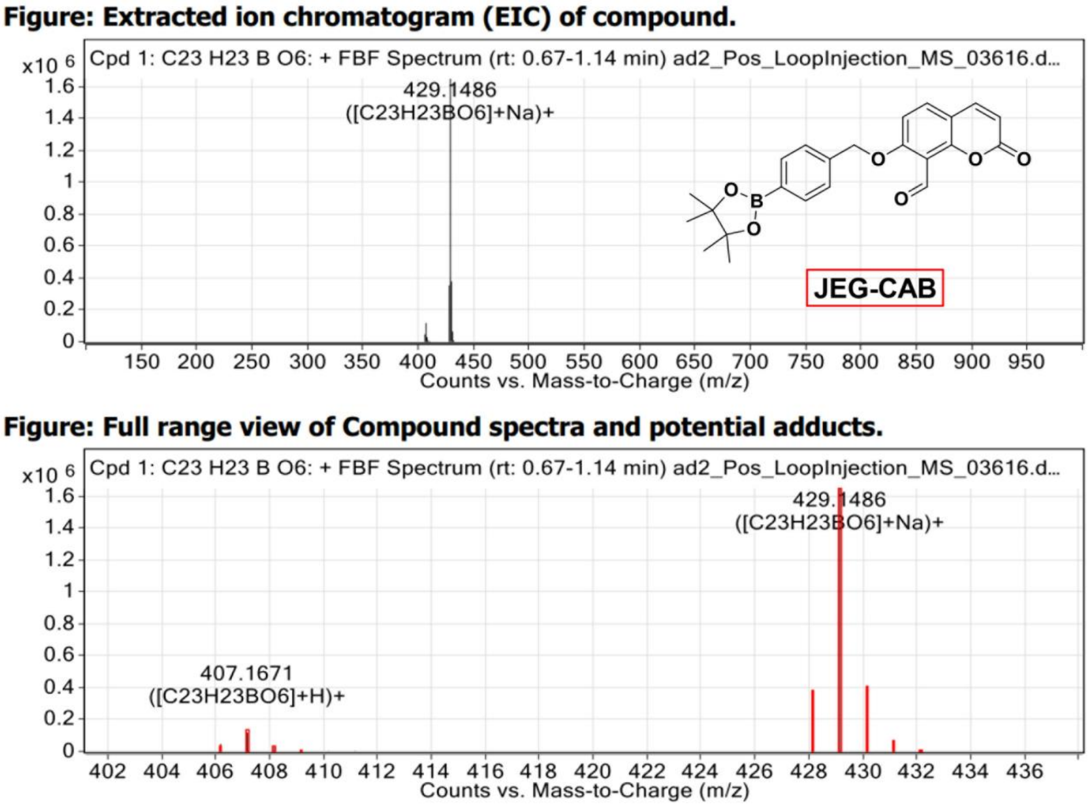


**Figure S19.** HRMS spectrum of **JEG-CAB** before the addition of analyte.

**Compound Table**

| Compound Label | RT (min) | Observed mass (m/z) | Neutral observed mass (Da) | Theoretical mass (Da) | Mass error (ppm) | Isotope match score (%) |
|---|---|---|---|---|---|---|
| Cpd 1: C10 H6 O4 | 3.83 | 189.0188 | 190.0260 | 190.0266 | -3.06 | 97.69 |

Mass errors of between -5.00 and 5.00 ppm with isotope match scores above 60% are considered confirmation of molecular formulae

**Figure: Extracted ion chromatogram (EIC) of compound.**

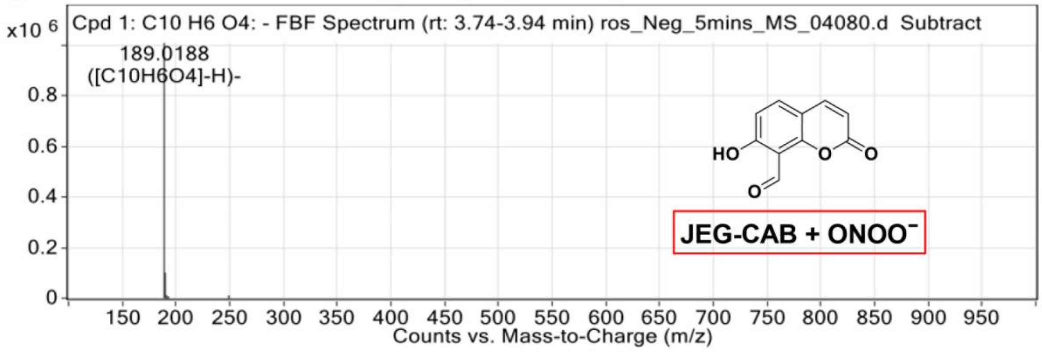


**Figure: Full range view of Compound spectra and potential adducts.**

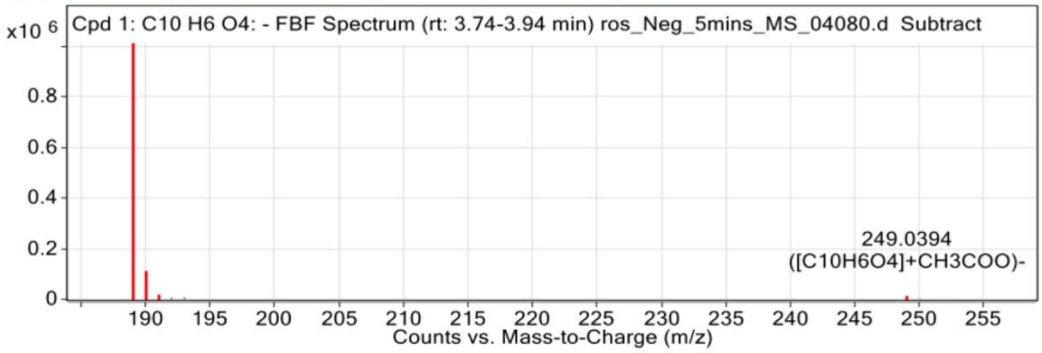


**Figure S20.** LC-MS spectrum of **JEG-CAB + ONOO$^-$**.

**Compound Table**

| Compound Label | RT (min) | Observed mass (m/z) | Neutral observed mass (Da) | Theoretical mass (Da) | Mass error (ppm) | Isotope match score (%) |
|---|---|---|---|---|---|---|
| Cpd 1: C14 H13 N O5 S | 0.60 | 308.0589 | 307.0510 | 307.0514 | -1.47 | 98.96 |

Mass errors of between -5.00 and 5.00 ppm with isotope match scores above 60% are considered confirmation of molecular formulae

**Figure: Extracted ion chromatogram (EIC) of compound.**

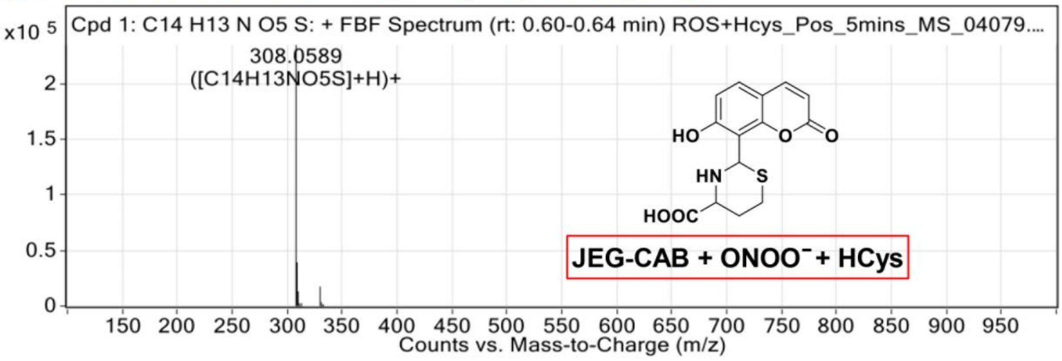


**Figure: Full range view of Compound spectra and potential adducts.**

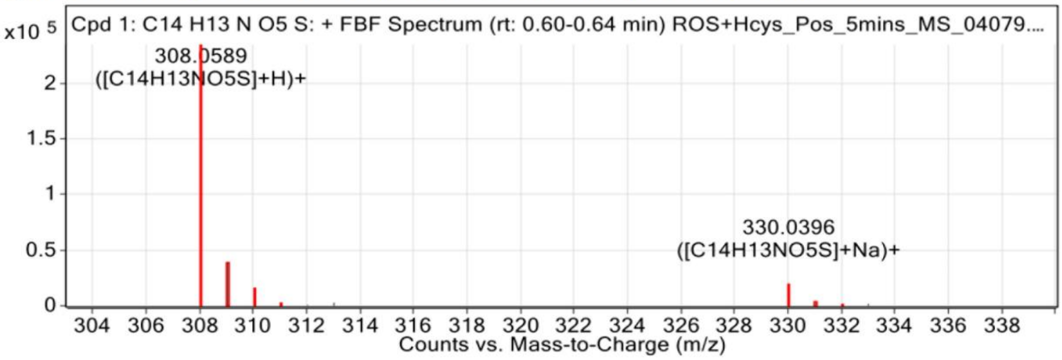


**Figure S21.** LC-MS spectrum of **JEG-CAB + ONOO$^-$ + HCys** followed by addition of **HCys**.

## 7. Mass spec analysis of JEG-CAN

LC-MS spectra were recorded on an Agilent 1200 series LC coupled to an Advion compact mass spec expression. LC-MS experiments were performed in order to determine the detection mechanism for the dual selective sensing strategy with **JEG-CAN** (10 μM) in the presence of 0.1 mM NADPH and 4 μg/mL nitroreductase (NTR) and 0.5 mM homocysteine in PBS buffer (pH 7.40, containing 1% DMSO). Experiments were performed after 2 hours of incubation with NTR and 1 hour with HCys.

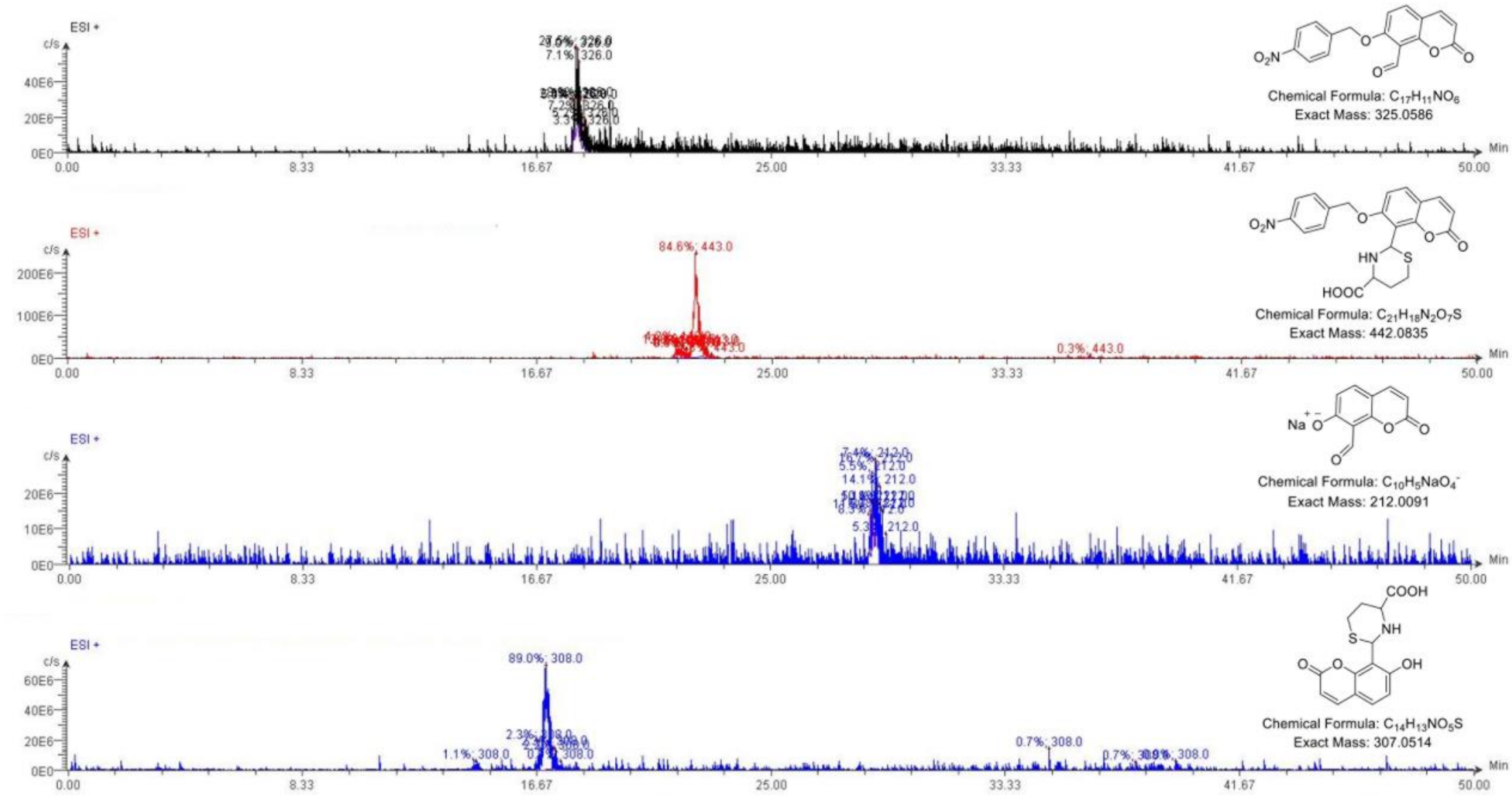


**Figure S22.** LC-MS extracted ion chromatograms of (a) **JEG-CAN**, (b) **JEG-CAN + HCys**, (c) **JEG-CAN +NADPH + NTR**, (d) **JEG-CAN + HCys + NADPH + NTR**.

# 8. Experimental

## 2-oxo-2*H*-chromen-7-yl acetate (2)

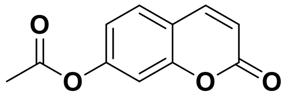

Umbelliferone (4 g, 22.2 mmol) and acetic anhydride ($Ac_2O$) (30 mL) was heated to 140 ºC and stirred for 2 h. The solution was then cooled to r.t. and the solvent was removed *in vacuo* to afford the title compound **2** as a brown powder. The product was used without further purification. M.p. 143-146 ºC. $^1$H NMR (500 MHz, $CDCl_3$) δ 7.68 (d, *J* = 9.6 Hz, 1H), 7.48 (d, *J* = 8.4 Hz, 1H), 7.11 (d, *J* = 2.2 Hz, 1H), 7.05 (dd, *J* = 8.4, 2.2 Hz, 1H), 6.39 (d, *J* = 9.6 Hz, 1H), 2.34 (s, 3H). $^{13}$C NMR (125 MHz, $CDCl_3$) δ 168.6, 160.3, 154.7, 153.4, 142.8, 128.5, 118.3, 116.1, 110.4, 21.1. IR (thinfilm) $\nu_{max}$ (cm$^{-1}$): 1732.20 (C=O), 1619.69 (C=O). HRMS (ES$^+$): calc. for $C_{11}H_8O_4$ [M+H]$^+$ 205.0495, found 205.049.

## 7-hydroxy-2-oxo-2*H*-chromene-8-carbaldehyde (CAH)

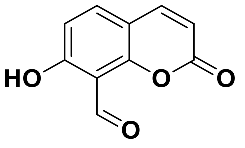

**2** (1.5 g, 7.35 mmol) was dissolved in trifluoroacetic acid (10 mL) at 0 °C and hexamethylenetetramine (1.5 g, 10.7 mmol) was added. The mixture was heated to reflux overnight and the solvent was then removed *in vacuo*. $H_2O$ (30 mL) was then added and the mixture was then heated to 60 °C for 30 min then cooled to r.t. Upon cooling, a yellow precipitate formed that was collected *via* filtration to yield 7-hydroxy-2-oxo-2*H*-chromene-8-carbaldehyde (**CAH**) (0.96 g, 69%). M.p. 151-152 °C. $^1$H NMR (500 MHz, DMSO-*d*$_6$) δ 10.40 (s, 1H), 7.99 (d, *J* = 9.6 Hz, 1H), 7.84 (d, *J* = 8.7 Hz, 1H), 6.93 (d, *J* = 8.7 Hz, 1H), 6.34 (d, *J* = 9.6 Hz, 1H). $^{13}$C NMR (125.7 MHz, DMSO-*d*$_6$) δ 191.22, 164.33, 159.51, 156.09, 144.91, 136.65, 114.35, 112.98, 111.57, 109.62. IR (thinfilm) $\nu_{max}$ (cm$^{-1}$): 3076.23 (OH), 1726.43 (C=O), 1597.58 (C=O).

**2-oxo-7-((4-(4,4,5,5-tetramethyl-1,3,2-dioxaborolan-2-yl)benzyl)oxy)-2*H*-chromene-8-carbaldehyde (JEG-CAB)**

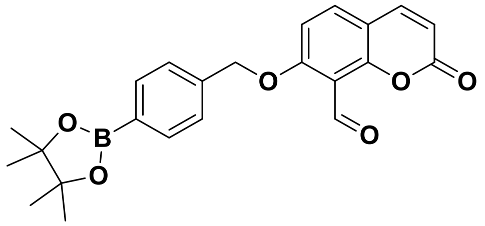

**CAH** (100 mg, 1.05 mmol) was dissolved in DMF (5 mL) and $K_2CO_3$ (215 mg, 3.15 mmol) was added while stirring. 4-bromomethylphenylboronic acid pinacol ester (343 mg, 1.157 mmol) was then added and the reaction was stirred for 4 h at r.t. When complete, the reaction mixturewas poured into $H_2O$ (50 mL) and stirred for 10 mins. The precipitate was filtered to yield a yellow solid, which was purified by silica column chromatography (40 % EtOAc/petroleum ether) to yield the final compound **JEG-CAB** (217 mg, 51%). M.p. 212-216 °C. $^1$H NMR (500 MHz, $CDCl_3$) δ 10.70 (s, 1H), 7.83 (d, $J$ = 8.0 Hz, 2H), 7.61 (d, $J$ = 9.6 Hz, 1H), 7.55 (d, $J$ = 8.8 Hz, 1H), 7.44 (d, $J$ = 7.9 Hz, 2H), 6.93 (d, $J$ = 8.8 Hz, 1H), 6.31 (d, $J$ = 9.6 Hz, 1H), 1.34 (s, 12H). $^{13}$C NMR (125.7 MHz, $CDCl_3$) δ 186.66, 162.24, 159.39, 155.78, 142.87, 138.23, 135.22, 133.81, 126.04, 114.22, 113.28, 112.80, 109.60, 83.91, 71.13, 53.40, 24.84; IR (thinfilm) $\nu_{max}$ (cm$^{-1}$): 1720.03 (C=O), 1690.84 (C=O). HRMS (ES$^+$): calc. for $C_{23}H_{23}BO_6$ [M+Na]$^+$ 429.1462, found 429.1486.

**7-((4-nitrobenzyl)oxy)-2-oxo-2H-chromene-8-carbaldehyde (JEG-CAN)**

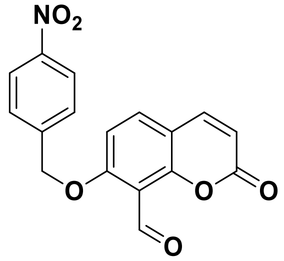

**CAH** (100 mg, 1.05 mmol) was dissolved in DMF (5 mL) and $K_2CO_3$ (215 mg, 3.15 mmol) was added while stirring. 4-nitrobenzyl bromide (249 mg, 1.157 mmol) was then added and the reaction was stirred for four hours at r.t. When complete, the reaction mixture was poured into water (50 mL) and stirred for 10 minutes. The mixture was filtered to yield a yellow solid. This solid was recrystallised in MeOH/DCM to yield pure **JEG-CAN** (161 mg, 49%). $^1$H NMR (500 MHz, DMSO-$d_6$) δ 10.55 (s, 1H), 8.31 – 8.25 (m, 2H), 8.03 (d, $J$ = 9.6 Hz, 1H), 7.96 (d, $J$ = 8.8 Hz, 1H), 7.80 (d, $J$ = 8.8 Hz, 2H), 7.29 (d, $J$ = 8.9 Hz, 1H), 5.53 (s, 2H). $^{13}$C NMR (125 MHz, DMSO-$d_6$) δ 187.18, 162.01, 159.61, 155.05, 147.58, 144.61, 144.28, 135.59, 128.46, 124.12, 114.01, 113.42, 112.76, 110.51, 69.85. M.p. 238-241 °C. I.R (thinfilm) $\nu_{max}$ (cm$^{-1}$): 1724.05 (C=O), 1685 (C=O), 1513 (N-O). HRMS (ES$^+$): calc. for $C_{17}H_{11}NO_6$ [M+H]$^+$ 326.0659 , found 326.0695.

## 9. NMR Spectra

### 2-oxo-2*H*-chromen-7-yl acetate (2)

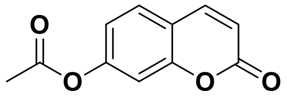

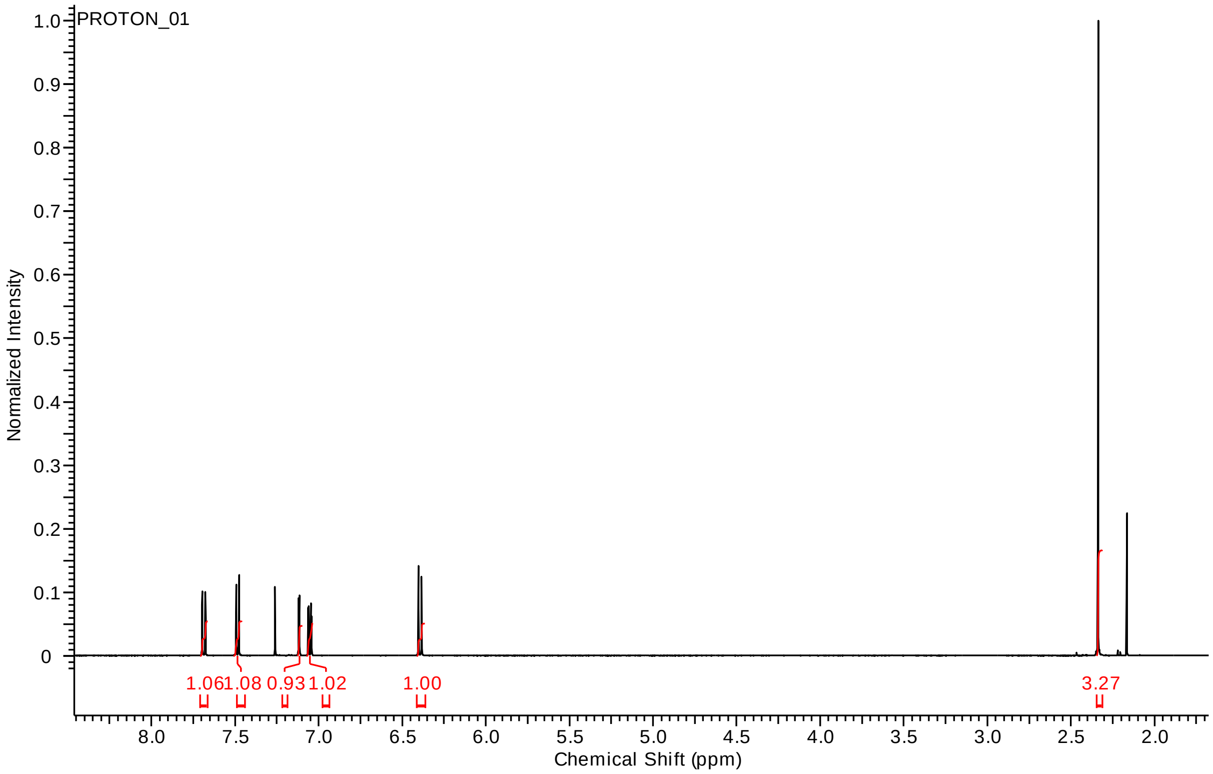


**Figure S23.** $^{1}$H NMR of compound **2**.

## 2-oxo-2*H*-chromen-7-yl acetate (2)

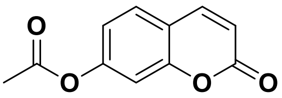

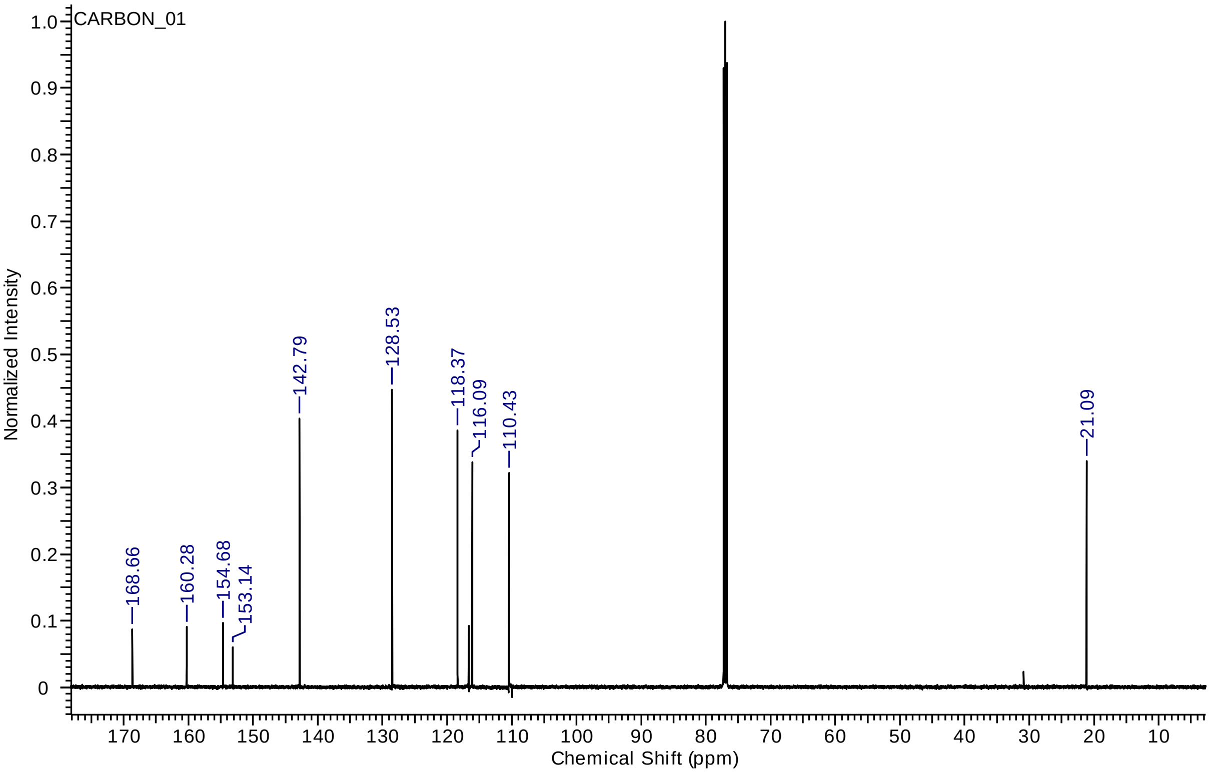


**Figure S24.** $^{13}C$ NMR of compound **2**.

## 7-hydroxy-2-oxo-2*H*-chromene-8-carbaldehyde (CAH)

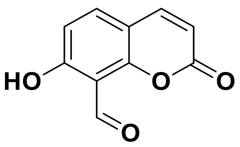

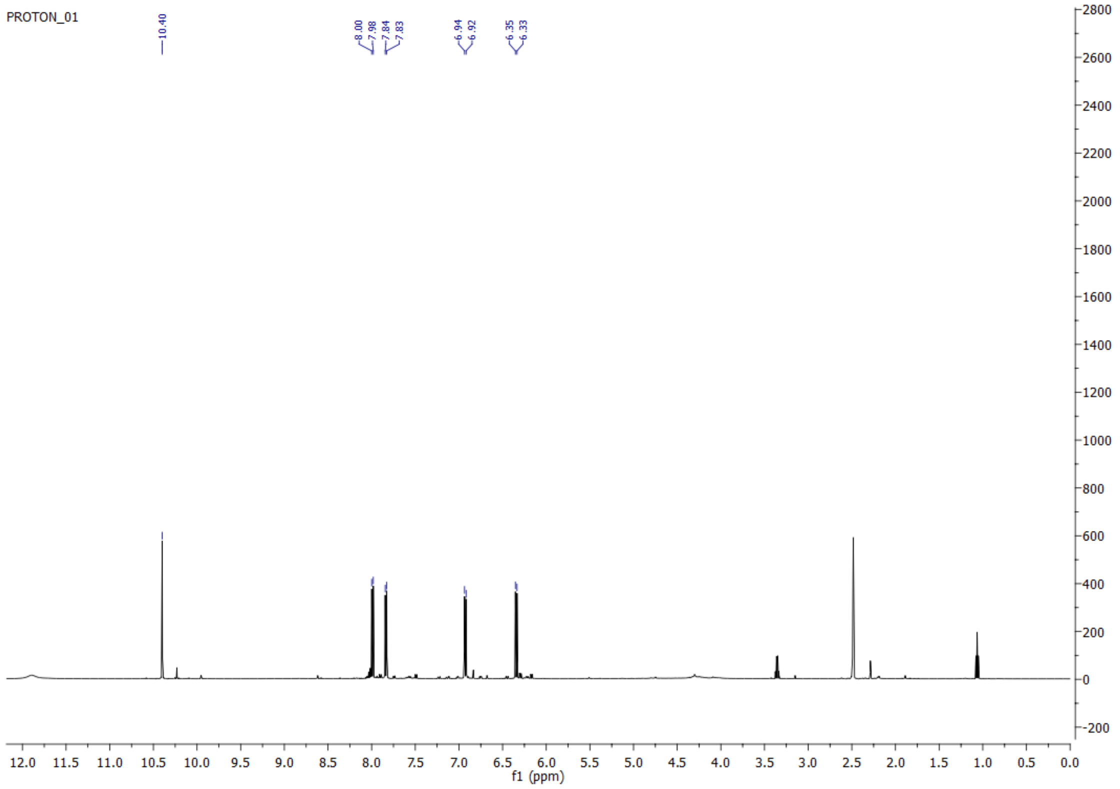


**Figure S25.** $^{1}$H NMR of compound **CAH**.

**7-hydroxy-2-oxo-2*H*-chromene-8-carbaldehyde (CAH)**

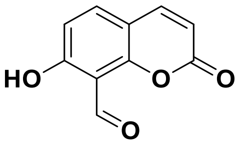

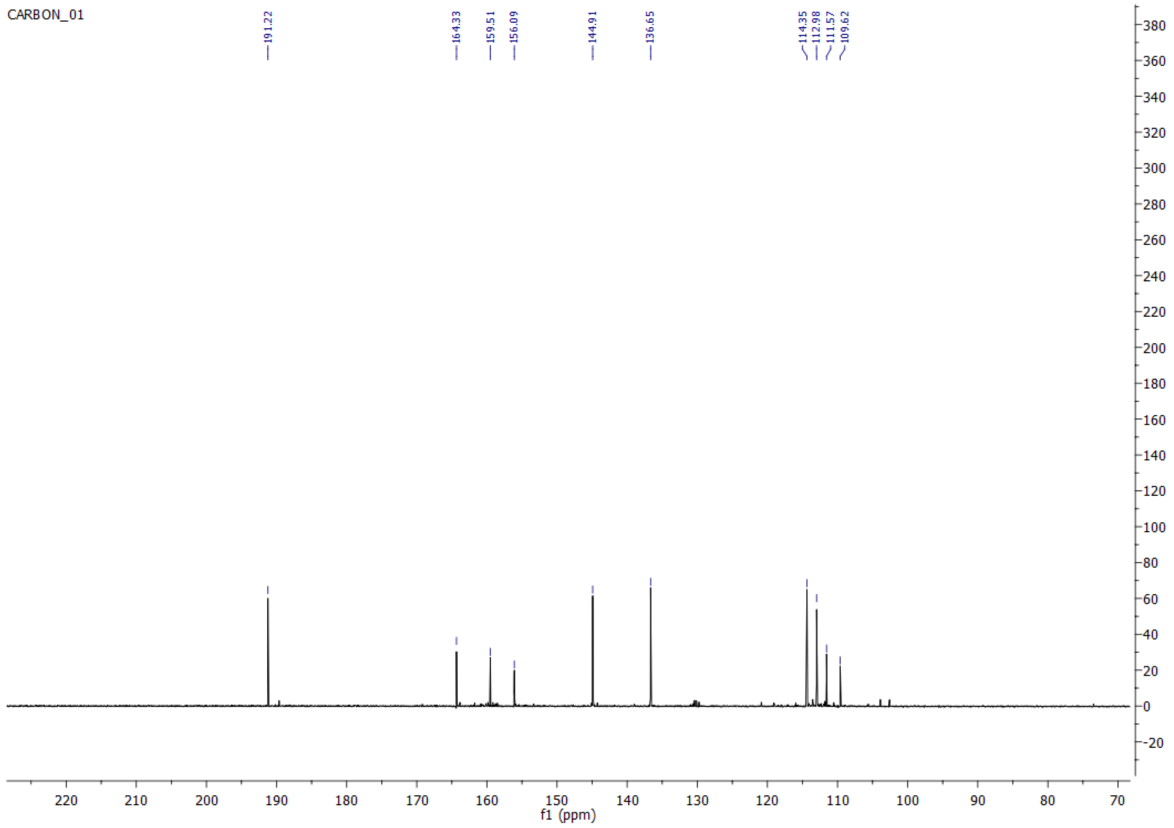


**Figure S26.** $^{13}$C NMR of compound **CAH**.

**2-oxo-7-((4-(4,4,5,5-tetramethyl-1,3,2-dioxaborolan-2-yl)benzyl)oxy)-2*H*-chromene-8-carbaldehyde (JEG-CAB)**

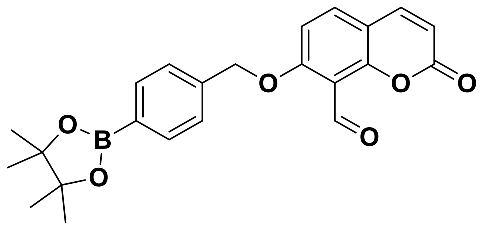

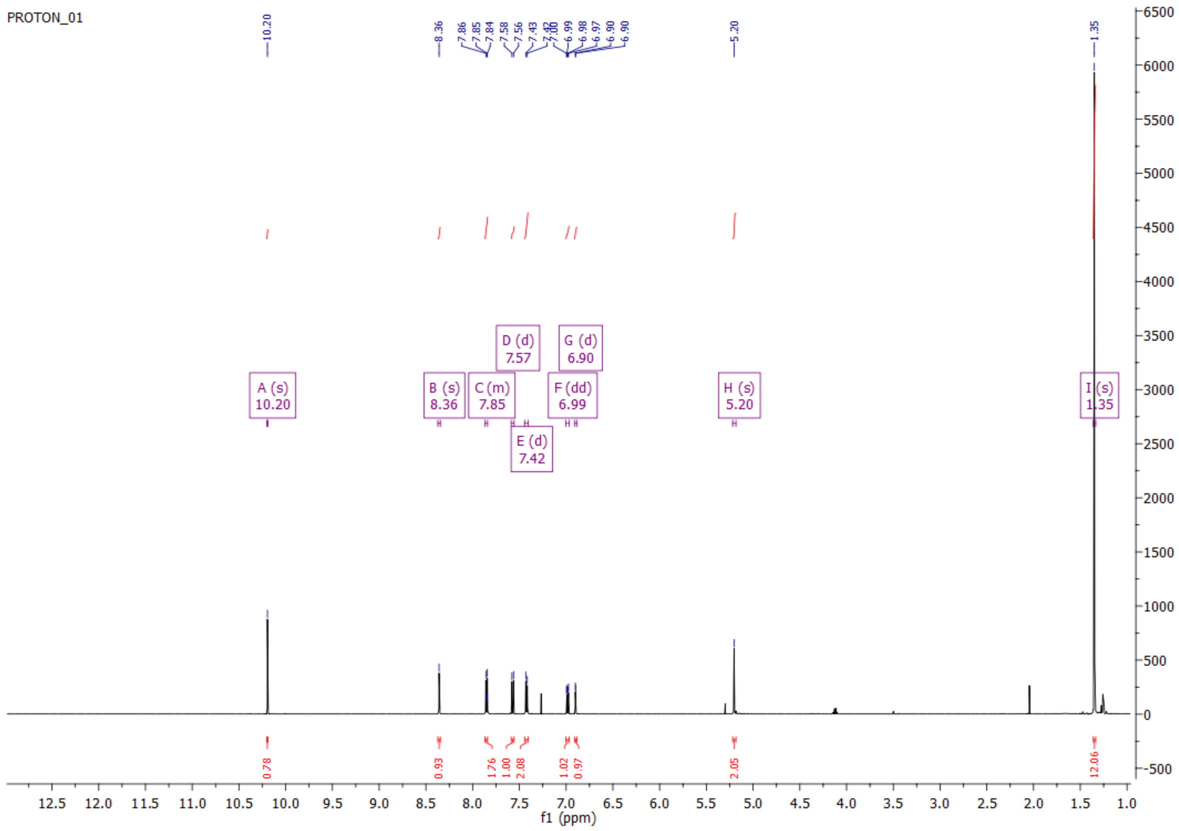


**Figure S27.** $^{1}H$ NMR of probe **JEG-CAB**.

**2-oxo-7-((4-(4,4,5,5-tetramethyl-1,3,2-dioxaborolan-2-yl)benzyl)oxy)-2*H*-chromene-8-carbaldehyde (JEG-CAB)**

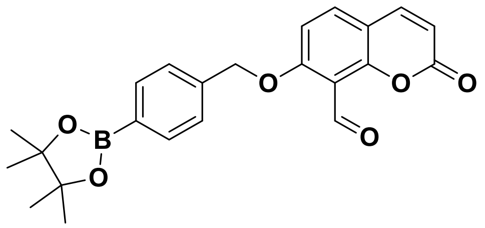

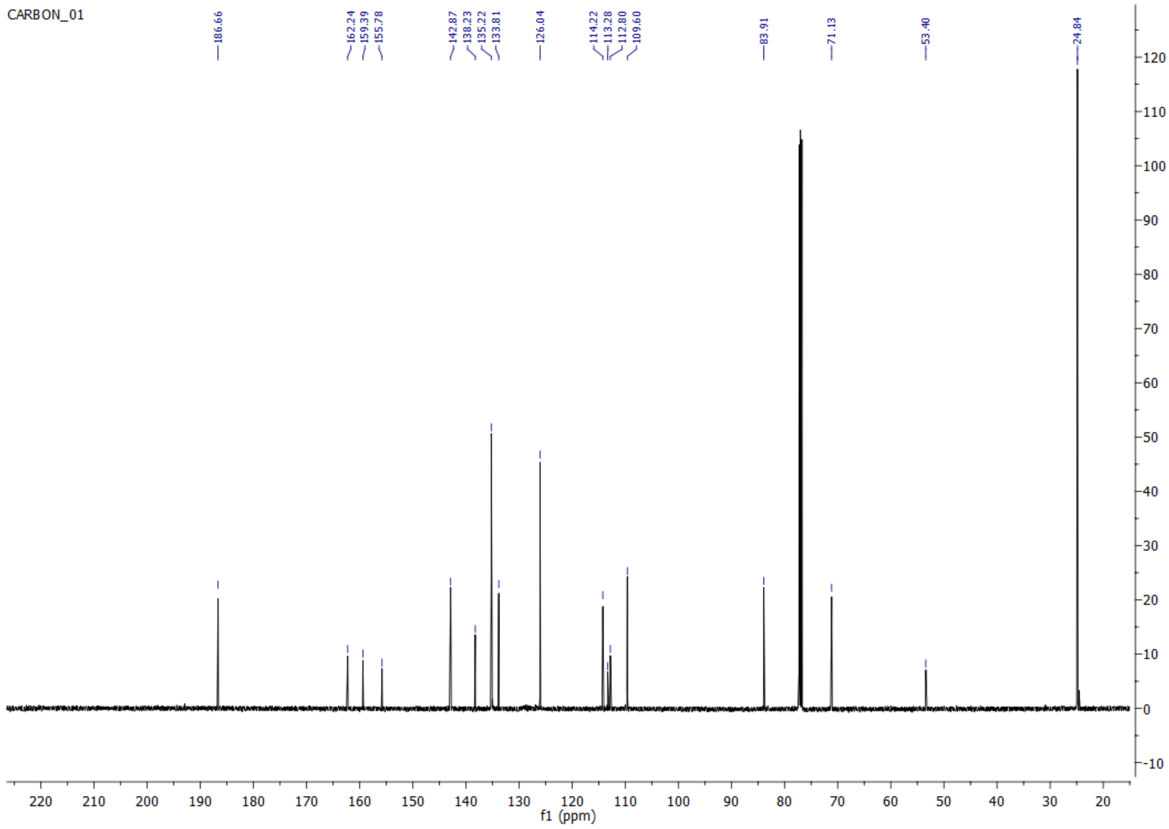


**Figure S28.** $^{13}$C NMR of probe **JEG-CAB**.

**7-((4-nitrobenzyl)oxy)-2-oxo-2H-chromene-8-carbaldehyde (JEG-CAN)**

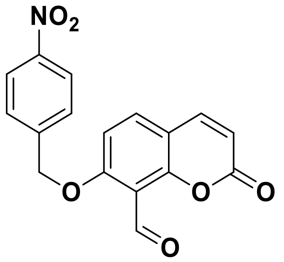


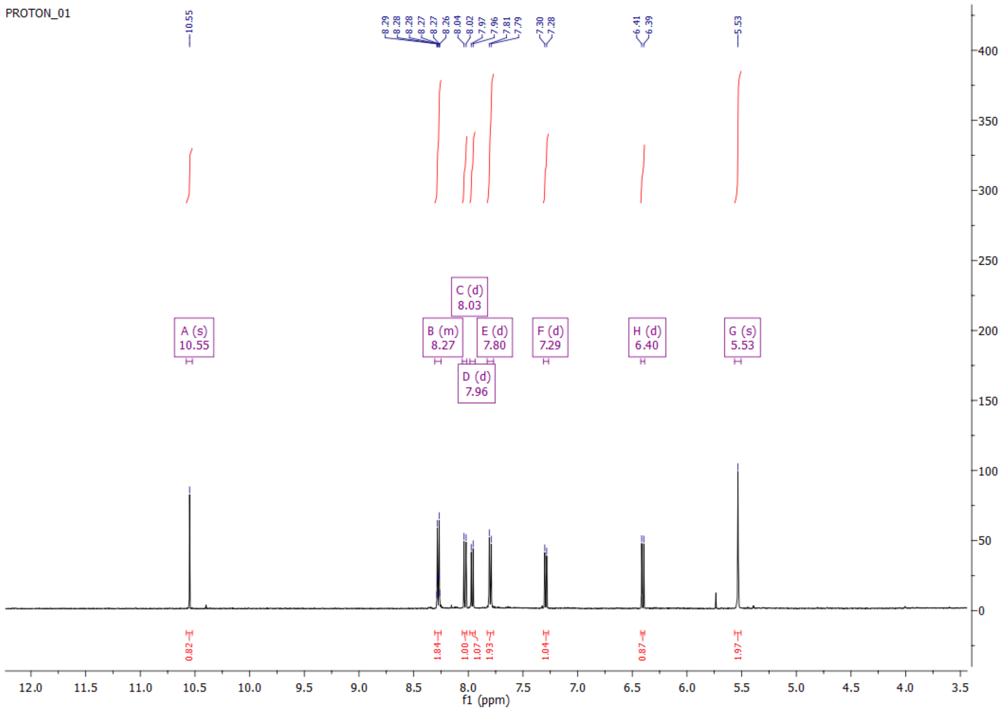


**Figure S29.** $^{1}$H NMR of probe **JEG-CAN**.

**7-((4-nitrobenzyl)oxy)-2-oxo-2H-chromene-8-carbaldehyde (JEG-CAN)**

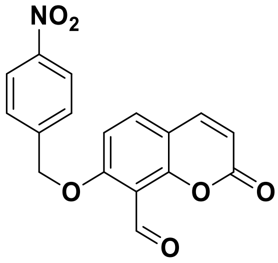


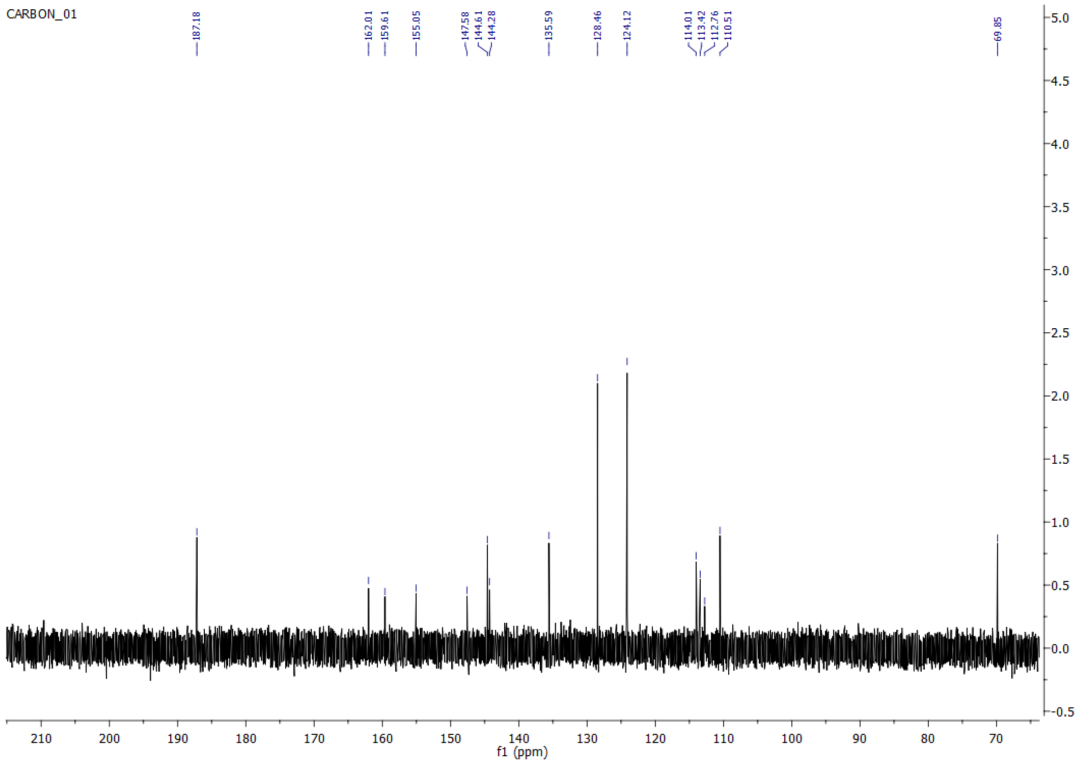


**Figure S30.** $^{13}$C NMR of probe **JEG-CAN**.

## 10. References


1. K.-S. Lee, T.-K. Kim, J. H. Lee, H.-J. Kim and J.-I. Hong, *Chem. Commun*., **2008**, 6173.


## 11 Author contributions

Luling Wu – wrote the manuscript, synthesized probes and carried out fluorescence experiments.
Jordan E. Gardiner – assisted Luling throughout the project in the synthesis of probes and fluorescence experiments.
Lokesh K. Kumawat – Carried out the nitroreductase experiments.
Hai-Hao Han – Carried out the cellular experiments on **JEG-CAB**.
Ruiying Guo – Carried out the cellular experiments on **JEG-CAN**.
Xin Li – Supervisor of Ruiying Guo.
Adam C. Sedgwick – conceived the idea and supervised Luling Wu and Jordan E. Gardiner.
Robert B. P. Elmes – Supervisor of Lokesh K. Kumawat.
Xiao-Peng He – Supervisor of Hai-Hao Han.
Steven D. Bull – Supervisor of Luling Wu and Jordan E. Gardiner.
Tony D. James – Lead Supervisor developed the idea with Adam C. Sedgwick and wrote the manuscript with Luling Wu.
